# Direct measurement of the thermoelectric properties of electrochemically deposited Bi_2_Te_3_ thin films

**DOI:** 10.1038/s41598-020-74887-z

**Published:** 2020-10-21

**Authors:** Jose Recatala-Gomez, Pawan Kumar, Ady Suwardi, Anas Abutaha, Iris Nandhakumar, Kedar Hippalgaonkar

**Affiliations:** 1 Institute of Materials Research and Engineering, Agency for Science Technology and Research, #08-03, 2 Fusionopolis Way, Innovis, Singapore, 138634 Singapore; 2 Department of Chemistry, University of Southampton, University Road, Highfield, Southampton, SO17 1BJ UK; 3 School of Material Science and Engineering, Nanyang Technological University, Singapore, 639798 Singapore; 4 Qatar Environment and Energy Research Institute, Hamad Bin Khalifa University, Qatar Foundation, Doha, 34110 Qatar

**Keywords:** Thermoelectrics, Electrochemistry

## Abstract

The best known thermoelectric material for near room temperature heat-to-electricity conversion is bismuth telluride. Amongst the possible fabrication techniques, electrodeposition has attracted attention due to its simplicity and low cost. However, the measurement of the thermoelectric properties of electrodeposited films is challenging because of the conducting seed layer underneath the film. Here, we develop a method to directly measure the thermoelectric properties of electrodeposited bismuth telluride thin films, grown on indium tin oxide. Using this technique, the temperature dependent thermoelectric properties (Seebeck coefficient and electrical conductivity) of electrodeposited thin films have been measured down to 100 K. A parallel resistor model is employed to discern the signal of the film from the signal of the seed layer and the data are carefully analysed and contextualized with literature. Our analysis demonstrates that the thermoelectric properties of electrodeposited films can be accurately evaluated without inflicting any damage to the films.

## Introduction

Thermoelectric (TE) materials can directly convert heat into electricity. The canonical TE material is described as a “*phonon-glass electron-crystal*”: a material that has the electrical properties of a crystal but the thermal properties of a glass^1^. This ideal situation can only be achieved through the optimisation of the conflicting properties embedded in the dimensionless figure-of-merit, *zT* = S^2^σT/(κ_e_ + κ_ph_), where S is the Seebeck coefficient, σ is the electrical conductivity, κ_e_ is the electronic thermal conductivity, κ_ph_ is the phonon thermal conductivity and T is the temperature^2^. The best material for room temperature applications is bismuth telluride (Bi_2_Te_3_), which has been synthetized employing a plethora of methods, such as co-evaporation^3,4^, molecular beam epitaxy (MBE)^5^, Metal Organic Chemical Vapour Deposition (MOCVD)^6^, pulsed layer deposition (PLD)^7^ and hydrothermal synthesis^8^. Among them, the electrodeposition technique offers high deposition rates and scalability, as well as the ability to tune multiple parameters for the desired output^9^. In addition, multiple materials can be electrodeposited, such as antimonides^10^, oxides^11^, metal–organic frameworks^12^ and nitrides, among others^13^. Many electrolytes have been utilised for the electrodeposition of bismuth telluride but undoubtedly, the electrodeposition from aqueous-acidic electrolytes is the most widespread^14^. However, TE measurement of electrodeposited thin films has been extremely challenging due to the metallic seed layer underneath the electrodeposited film^15,16^. Two approaches have been traditionally employed and are summarised in Supplementary Table I. The first approach is to eliminate the contribution of the seed layer by mechanically separating it from the electrodeposited thin film. The main issue with this approach is that the properties of thin films are highly affected by stress and strain phenomena induced during the transfer, as they are directly involved in the creation of defects. These defects will in turn change the electrical conductivity and Seebeck coefficient. Hence, the measured properties will not correspond to the as-synthetized film^9^. The second approach is to measure the TE properties directly on top of the seed layer. This approach has proven to be troublesome or inaccurate thus far, as the seed layer can short-circuit the voltage along the layer during the measurement of the Seebeck coefficient and/or overestimate the value of the electrical conductivity^2^.

Our work demonstrates that the TE properties of electrodeposited films can indeed be accurately measured, building upon the second approach. We leverage upon the measurement technique developed by Kumar et al. and extend it to apply to electrochemically fabricated thin films^17^. For this purpose, bismuth telluride thin films have been electrodeposited onto indium tin oxide (ITO). The bismuth telluride films have been characterised in order to obtain a complete understanding of the morphology, composition and crystal structure and their impact on the TE properties. Thereafter, the temperature dependent TE properties of both ITO seed layer and Bi_2_Te_3_ have been evaluated and the data thoroughly analysed. A proper benchmarking of the experimental data herein collected with the literature led to the conclusion that the electrodeposited films can be measured in an accurate manner without damages.

## Results

**Figure 1:**
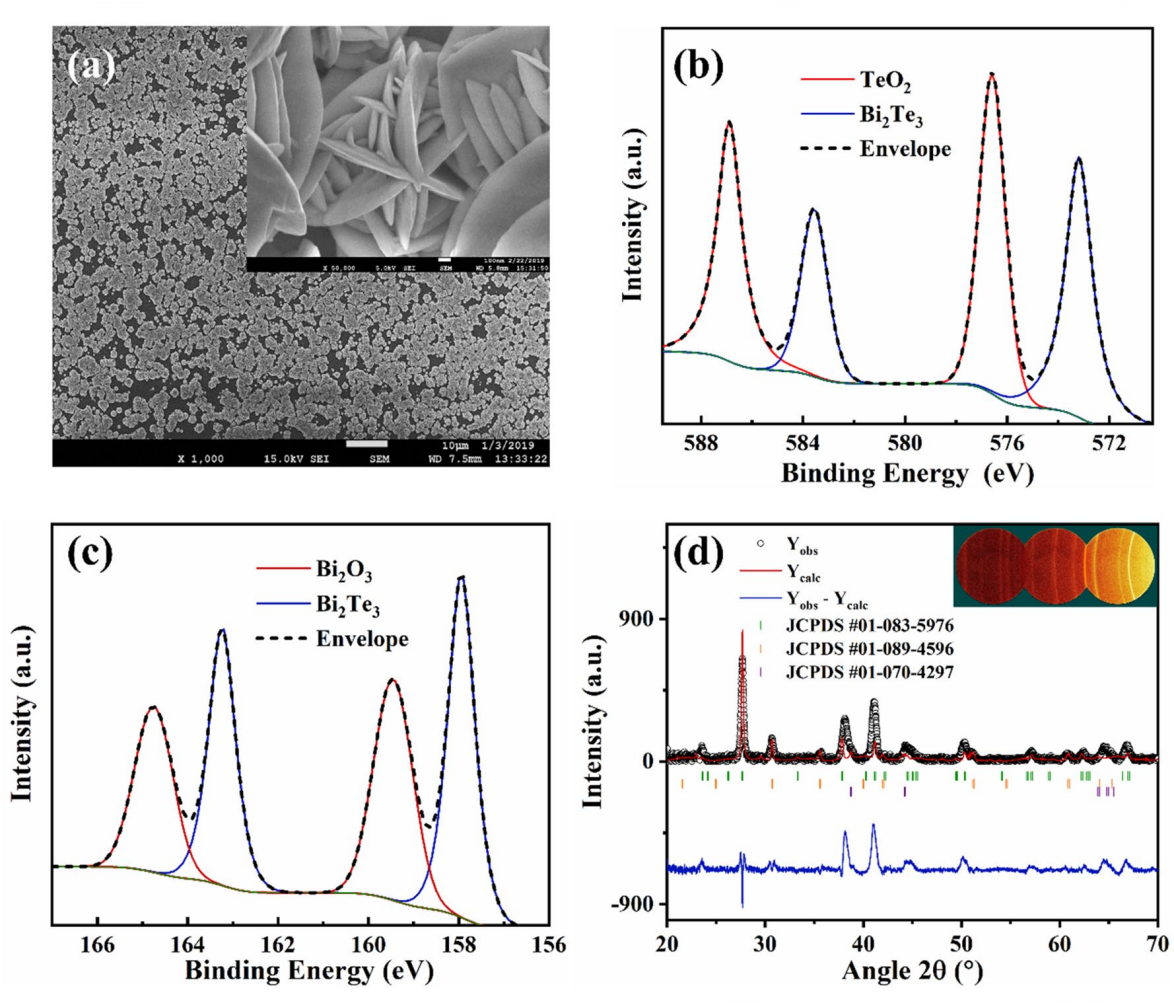
(**a**) Typical top view SEM image of an electrodeposited bismuth telluride thin film. Scale bar is 10 μm (inset: SEM image showing the microstructure. Scale bar is 100 nm), (**b**) core-level Te 3*d* spectrum, (**c**) core-level Bi 4*f* spectrum and (**d**) Typical diffraction patterns for bismuth telluride films (Y_obs_, black circles) with the corresponding Rietveld refinement (Y_calc_, red line), the difference between experimental and modelled data (Y_obs_ − Y_calc_, blue line) and the respective Bragg positions of the different phases: Bi_2_Te_3_ (green), ITO (orange) and TeO_2_ (purple). Inset: Typical 2D XRD pattern collected for the films. The rings indicate the polycrystalline nature of the sample.

The morphology of the electrodeposited films was investigated by means of Scanning Electron Microscopy (SEM). A typical image for the electrodeposited film has been shown in Fig. 1a. Nodular morphology was observed in all films, which is expected since all were grown at the same deposition potential (see “Methods” and Supplementary Section 2). The features comprising the film have a cauliflower-like shape, characteristic of films electrodeposited at low overpotentials under mass transport limiting conditions^18^. The lower compactness of the films, especially if compared with films fabricated with other techniques (physical techniques like PLD or sputtering tend to render denser films) could indicate that the electrical conductivity will be lower than the electrical conductivities expected for denser films^9^. This may arise from the high number of grain boundaries in the film, which is expected to lower the mobility, in turn lowering the electrical conductivity^19^.

Energy-dispersive X-ray spectroscopy (EDX) was carried out to obtain the composition of the films. Spectra were recorded across three different areas of the films in order to make the measurement reliable. The composition of the samples is constant, deviated from the theoretical 40% atomic Bi and 60% atomic Te, corresponding to stoichiometric Bi_2_Te_3_. A summary of the results can be found in Table 1.

**Table 1:** Summary of elemental and structural parameters of the three electrodeposited bismuth telluride thin films (referred to as Sample 1, Sample 2 and Sample 3).

Sample	t (μm)	EDX at.%Bi	EDX at.%Te	XPS at.%Bi	XPS at.%Te	D_XRD_ (nm)
1	1.20 ± 0.08	41.09 ± 0.21	58.91 ± 0.21	43.97	56.03	33.10 ± 0.16
2	2.50 ± 0.04	38.90 ± 0.14	61.10 ± 0.14	40.52	59.48	24.33 ± 0.10
3	3.75 ± 0.03	41.33 ± 0.31	58.67 ± 0.31	43.80	56.20	21.21 ± 0.12

The thickness of the film (t) was determined by profilometry. Three measurements in different parts of the thin film were recorded and the standard deviation calculated  (see Supplementary Section 9 for details about error calculations). The crystallite size (D_XRD_) was calculated using the Scherrer equation (see Supplementary Section 3 for further details).

X-ray Photoelectron Spectroscopy (XPS) was used to investigate the oxidation state of the bonded elements as well as to further determine the composition of the electrodeposited films. Figure 1b and c shows the core-level of Te 3*d* and Bi 4*f* spectrum, respectively. The presence of the peaks at 573.15 eV and 583.5 eV in Fig. 1b and the peaks at 158.7 eV and 164 eV in Fig. 1c confirm that non-stoichiometric bismuth telluride (Bi_2_Te_3_) has been formed, in agreement with the EDX data. The extra peaks in both Fig. 1b and c indicate that a mixed phase comprising bismuth oxide and tellurium oxide has been formed, well along the lines of previous reports^20^. Interestingly, the Bi content is higher in the surface part (XPS, sampling depth ~ 10 nm) for two samples (see data for Sample 1 and Sample 3 in Table 1) than that of Te compared to the vice-versa in the bulk (EDX, sampling depth depends on accelerating voltage but it is in the ~ 2–3 μm range). These results were compared to previous studies and made us conclude that there is no strong, clear correlation between the electrochemical method and the higher content of atomic Bi in the surface of the film (measured by XPS) compared to higher content of atomic Te in the bulk of the film (measured by EDX). Hence, the only thing we can say conclusively is that both techniques indicate non-stoichiometry. Thus, we have attributed this discrepancy to the different sampling depth between both techniques, as several authors also concluded^14, 21–33^. The crystal structure of the electrodeposited films is investigated by means of x-ray diffraction (XRD). Figure 1d shows a typical diffraction pattern with the corresponding Rietveld refinement. The majority of peaks are fitted to the “Thellurobismutithe” file (JCPDS file n° 01-083-5976)^34^. Other peaks are assigned to the substrate, indium tin oxide (ITO, JCPDS file n° 01-089-4596)^35^ and tellurium oxide (TeO_2_, JCPDS file n° 01-070-4297)^36^. The low intensity of the peaks corresponding to TeO_2_ indicates that it is only present in the surface, which is in line with the results obtained during XPS analysis^20^. The crystallite size (D_XRD_) of the films are summarised in Table 1. A detailed analysis of the XPS spectra and the XRD pattern and crystallite size can be found in Supplementary Section 3.

**Figure 2:**
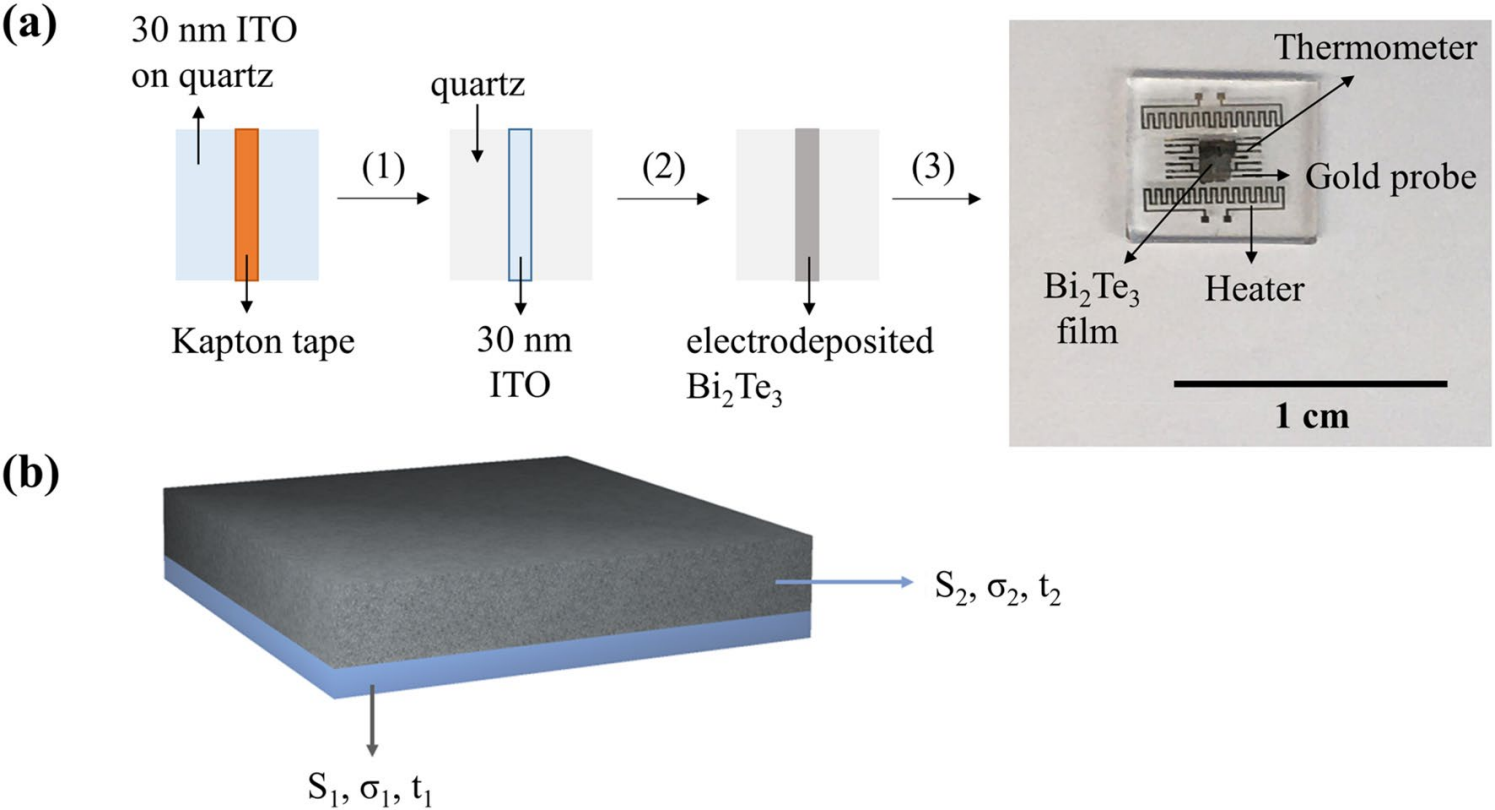
(**a**) Schematic representation of the fabrication workflow of the devices for TE measurements. First, the ITO area in which the bismuth telluride thin film will be electrodeposited is defined by masking with Kapton tape (represented by the orange stripe). Subsequently the unmasked ITO is etched out by immersion in a 6 M HCl solution (1), and only the defined ITO area for deposition is left. The second step (2) in the fabrication process is the electrodeposition of a bismuth telluride thin film on top of the ITO seed layer, purposely left out after etching. Finally, the device elements are evaporated (3): two thermometers, another two probes for resistivity measurement (labelled “Gold probe”) and two heaters (labelled “Heater”), all visible in the optical microscope image in (**a**). (**b**) Schematic representation of the two-band model deployed for the modelling of the ITO seed layer (with properties S_1_, σ_1_ and t_1_) and the electrodeposited film (with properties S_2_, σ_2_ and t_2_).

Finally, the device structure for TE measurements is fabricated containing two heaters, two resistance-based thermometers with four probe contacts, and two additional electrodes for four-probe electrical resistivity measurements (Fig. 2). Before proceeding to the measurement, the film is disconnected from the heaters to prevent electrical leakage. The temperature dependent in-plane Seebeck coefficient and electrical conductivity have been measured simultaneously^17^. The temperature dependent homemade thermoelectric measurement setup is designed inside a high-vacuum cryostat. The device is mounted on a 24-pin chip carrier using GE varnish. The electrical connections between the device and chip holder were made using a West Bond wire-bonder, threaded with aluminium wire.

A two-band model is applied to the seed layer, 30 nm of ITO (Material 1) and electrodeposited bismuth telluride (Material 2) thin film; samples with different thicknesses for Material 2 are fabricated. These two films are modelled as two resistors electrically connected in parallel (see Fig. 2b). Equations 1 and 2 describe the effective conductivity (σ_eff_) and effective Seebeck coefficient (S_eff_) measured for the parallel combination of seed layer and thin film^37^.

1 $${\sigma }_{eff}= \frac{{\sigma }_{1} {t}_{1}+ {\sigma }_{2} {t}_{2}}{{t}_{1}+ {t}_{2}}$$

2 $${S}_{eff}= \frac{{S}_{1}{\sigma }_{1}{t}_{1}+ {S}_{2}{\sigma }_{2}{t}_{2}}{{\sigma }_{1} {t}_{1}+ {\sigma }_{2} {t}_{2}}$$

where *S*_*1*_, *σ*_*1*_*, S*_*2*_, *σ*_*2*_ are the Seebeck coefficient and electrical resistivity of ITO and Bi_2_Te_3_, respectively. The thickness of the materials is represented by *t*_*1*_ and *t*_*2*_ and they correspond to ITO and Bi_2_Te_3_.

In order to obtain the electrical conductivity of the electrodeposited films, the materials must be chosen carefully. According to Eq. (1), if one of the materials has a much larger conductivity than the other one for similar thickness range (for instance, when the product ${\sigma }_{1} {t}_{1}$ is larger than ${\sigma }_{2} {t}_{2}$), the signal that will be read (*σ*_*eff*_) will be close to *σ*_*1*_. Hence, each layer’s electrical conductance should be of the same order of magnitude, although this is difficult in practice due to the metallic nature of seed layer and the semiconducting nature of the film. Regarding the ratio, ideally both materials should have similar range of thickness^37^. Thus, the chosen materials are ITO (Material 1) and Bi_2_Te_3_ (Material 2). ITO is a heavily doped semiconductor with high electrical conductivity, but lesser than that of common seed layers for electrodeposition (e.g., gold has a larger electrical conductivity, σ ~ 4.1 × 10^5^ S cm^–1^). In addition, the ITO seed layer sits on top of quartz. This material has been chosen as substrate because it has a low thermal conductivity thus ensuring the ability to generate a sufficient temperature gradient^17,38,39^. Material 2 can be any electrodeposited material. In principle, the seed layer is chosen by choosing the target material. This means that oxides such as antimony doped tin oxide (SnO_2_:Sb), which have slightly lower values of electrical conductivity (≈10^3^ S cm^–1^, depending on the fabrication technique) are more suitable for target materials that are less conductive, such as bismuth selenide. If Material 2 is a film even more insulating, for instance bismuth sulphide, an even more insulating oxide is to be utilized, such as amorphous indium and gallium doped zinc oxide (a-In-Ga-Zn–O, conductivities in the order of 10^3^–10^2^ S cm^–1^) in the case of a transparent conductive oxide or a semiconductor, such as phosphorus doped silicon (Si:P, with conductivities in the order of 10^2^ S cm^–1^)^40^. Of course, this will be also linked to the ability to electrodeposit Material 2 on Material 1, since electrodeposition requires good electrical contact between the two of them for the deposition current or voltage to be applied without impediment. We recommend the reader to take extra precaution because selecting the pair Materials 1–2 is very critical and of upmost importance in order to ensure a successful experiment and clear data interpretation.

**Figure 3:**
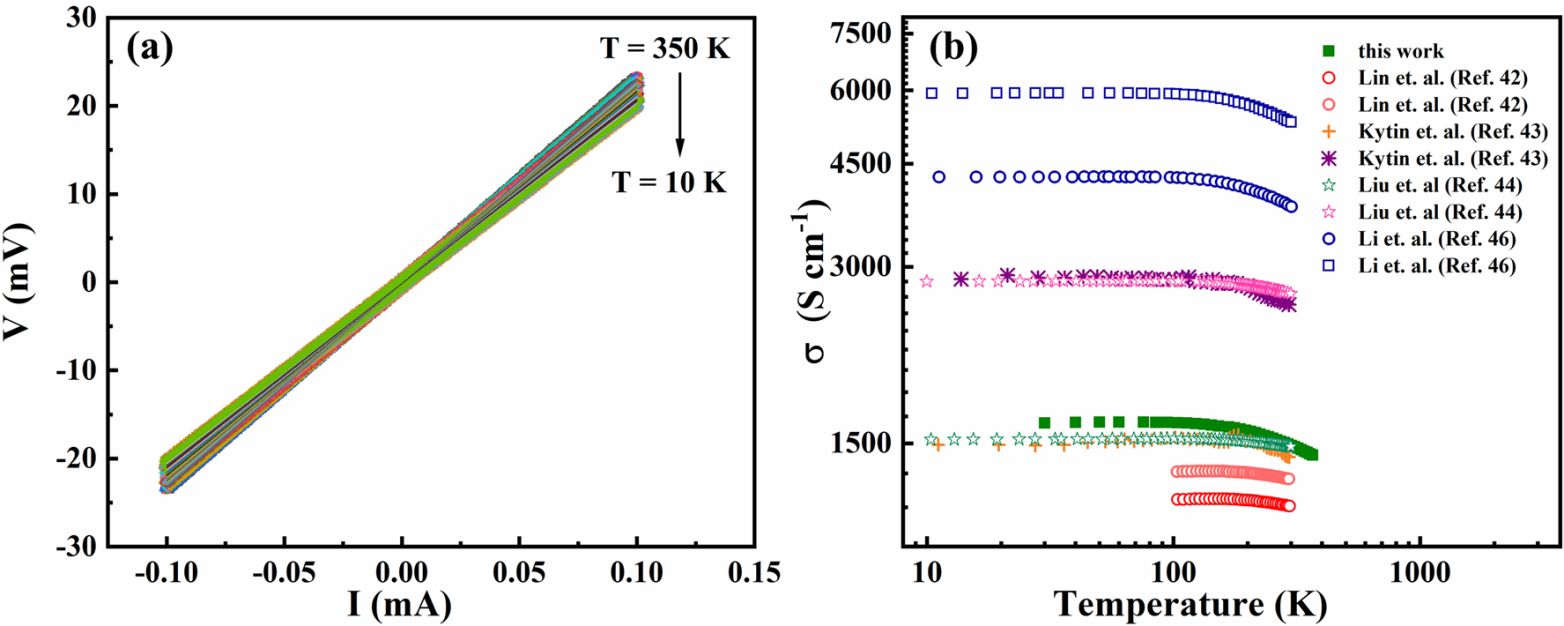
(**a**) Temperature dependent 4-probe I-V measurements (I-source, V-measured) for ITO (30 nm) and (**b**) Temperature dependent electrical conductivity of the ITO seed layer, compared with the literature.

First, the temperature dependent TE properties of the ITO seed layer were characterised. Figure 3a shows the I-V curves performed at different temperatures in a 4-probe arrangement. The voltage rises linearly with the current, demonstrating the ohmic nature of the contact^17^. Figure 3b compares the temperature dependent electrical conductivity of the ITO seed layer (σ_1_ in Eq. 1 and Eq. 2) with different literature values. As it can be seen, the values considerably differ from one author to others. This different values are attributed to the different carrier concentrations and different mobility of the ITO^41^. The crystallite size of the ITO films employed in this work was calculated using the Scherrer Equation, based on the XRD peak broadening of the (222) peak and is equal to 31.43 nm.

The carrier concentration of the ITO seed layer is determined by Hall measurements (see Supplementary Section 5) and equal to 4.42 × 10^21^ cm^−3^ at room temperature. The mobility was determined to be 2.11 cm^2^ V^−1^ s^−1^ at the lowest temperature recorded in this work (30 K). Larger crystallite sizes have been associated with higher mobility and therefore higher conductivity, which explains why the ITO measured in this work has larger electrical conductivity than the values reported by Lin et al.^42^ and some samples synthesised by Kytin et al.^43^ and Liu et al.^44^ (see Supplementary Section 5 for more details). The difference in conductivity with respect to the conductivity reported by Li et al. probably is due to the fact that they did not conduct a direct measurement of the carrier concentration by means of Hall measurement, but they calculated their carrier concentration, using a value of effective mass of m* = 0.4m_e_ from a Seebeck measurement. Hence, it could be possible that the carrier concentration differs from the reported theoretical values^45,46^.

**Figure 4:**
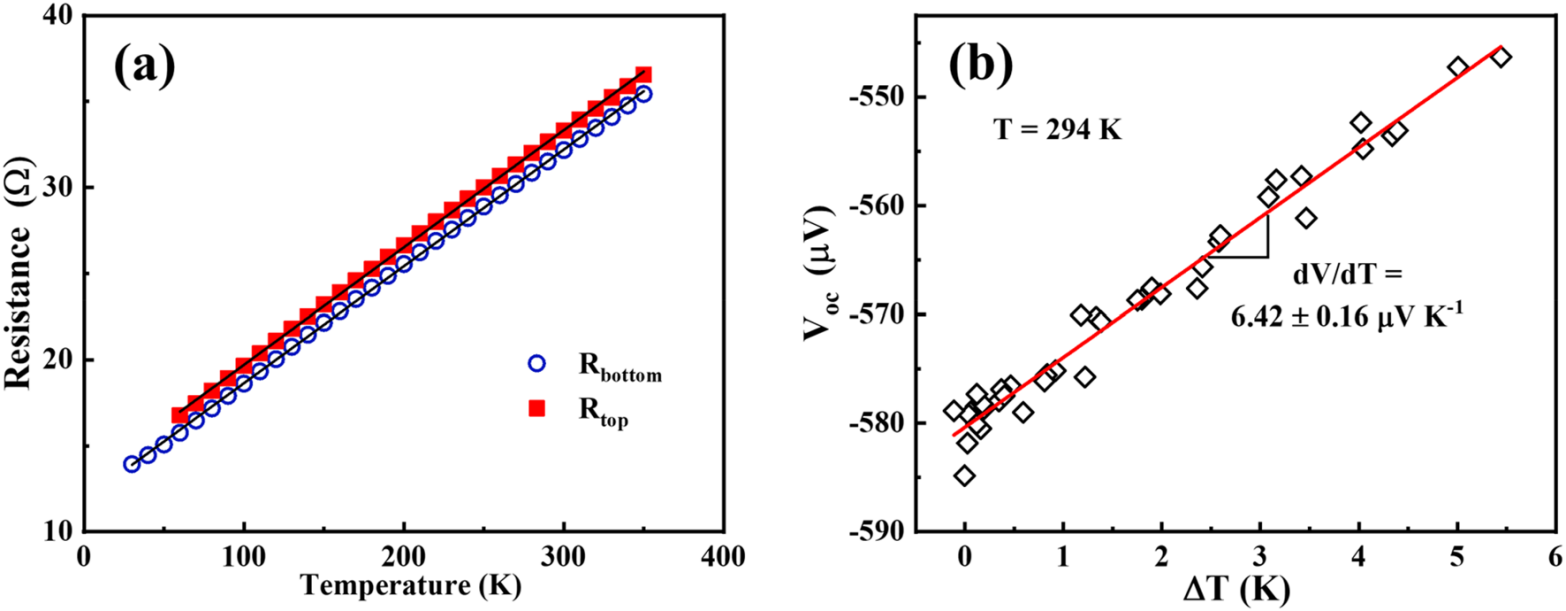
(**a**) Change in resistance of both thermometers (R_top_ is the resistance of hot thermometer and R_bottom_ is the resistance of cold thermometer) as a function of temperature for the ITO seed layer. The black lines correspond to linear fits R(T) = R_0_ + AT. For the cold thermometer, this equation is R(T) = 11.86 (± 0.042) + 0.068 (± 1.96 × 10^–4^)T and for the hot thermometer R(T) = 12.90 (± 0.054) + 0.0681 (± 2.45 × 10^–4^) T. (**b**) Open circuit voltage dependence with the temperature difference. The red line corresponds to a linear fit in order to obtain the Seebeck coefficient*.*

Next, calibration of the fabricated thermometers is performed: the correlation between the resistance of the thermometers and the temperature is evaluated (further discussion is presented in Supplementary Section 4), as depicted in Fig. 4a. A complete derivation of this procedure can be found elsewhere^17^*.* Fig. 4b shows the measured open-circuit voltage (V_oc_) as a function of temperature gradient (ΔT). A linear relation is observed as expected with slope of − 6.42 μV K^−1^. Considering the aluminium Seebeck coefficient of − 1.7 μV K^−1^ ^47^, the actual room temperature Seebeck coefficient of ITO was calculated using Supplementary Eq. S4 (see Supplementary Section 4) and found to be − 7.8 μV K^−1^. This is in line with the reported values of Seebeck coefficient for ITO^42,46,48^.

**Figure 5:**
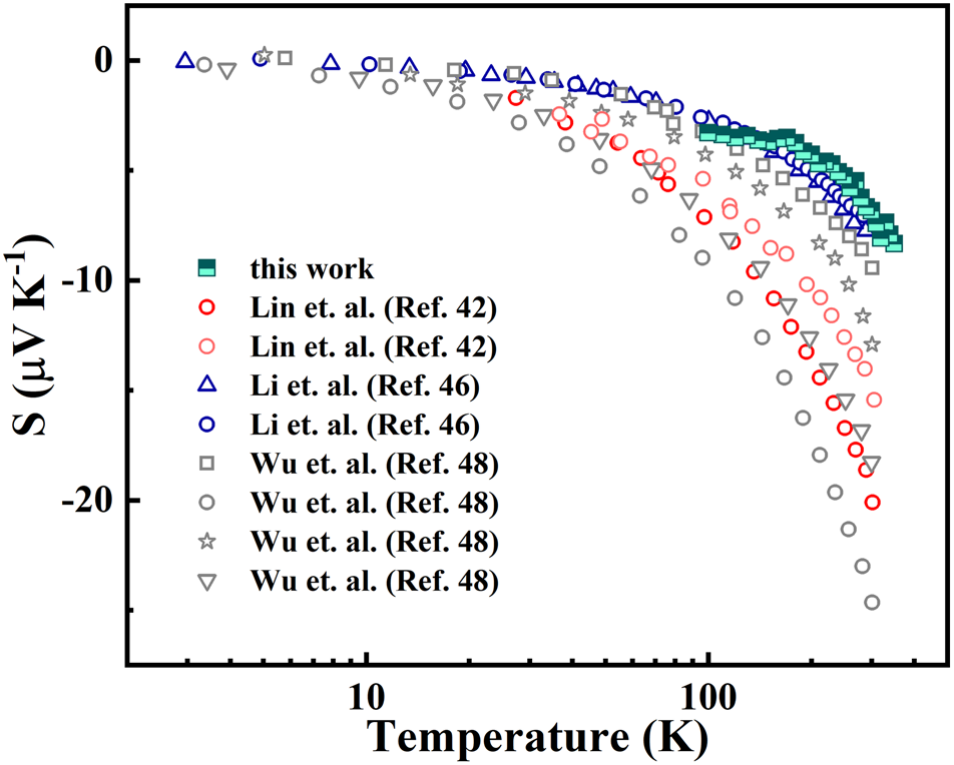
Temperature dependent Seebeck coefficient for the ITO seed layer (S_1_ in Eq. 2) benchmarked against previous reports.

The temperature dependent Seebeck coefficient for different ITO samples is compared in Fig. 5. As observed, the change of Seebeck with respect to the temperature follows a linear decreasing trend but the slope is not the same. The general expression for the full Boltzmann Transport Equation (BTE) for the Seebeck coefficient can be written as^2^:

3 $$S= -\frac{{k}_{B}}{q}\left[\eta -\frac{\left(r+\frac{5}{2}\right){F}_{r+\frac{3}{2}}(\eta )}{\left(r+\frac{3}{2}\right){F}_{r+\frac{1}{2}}(\eta )}\right]$$

where k_B_ is the Boltzmann constant, q is the elemental charge, η is the reduced Fermi energy, F(η) is the Fermi integral and r is the scattering parameter. From Eq. (3), in the degenerate limit, the Mott formula can be used to understand the trend where the Seebeck coefficient depends on the ratio m^*^/n^2/3^^2^. Thus, the change in the ratio is expected to be given by the change in carrier concentration for the different samples^14^. This change is the direct cause of the change in the slopes of the curves, but it is difficult to estimate since the other experiments did not report a carrier concentration.

**Figure 6:**
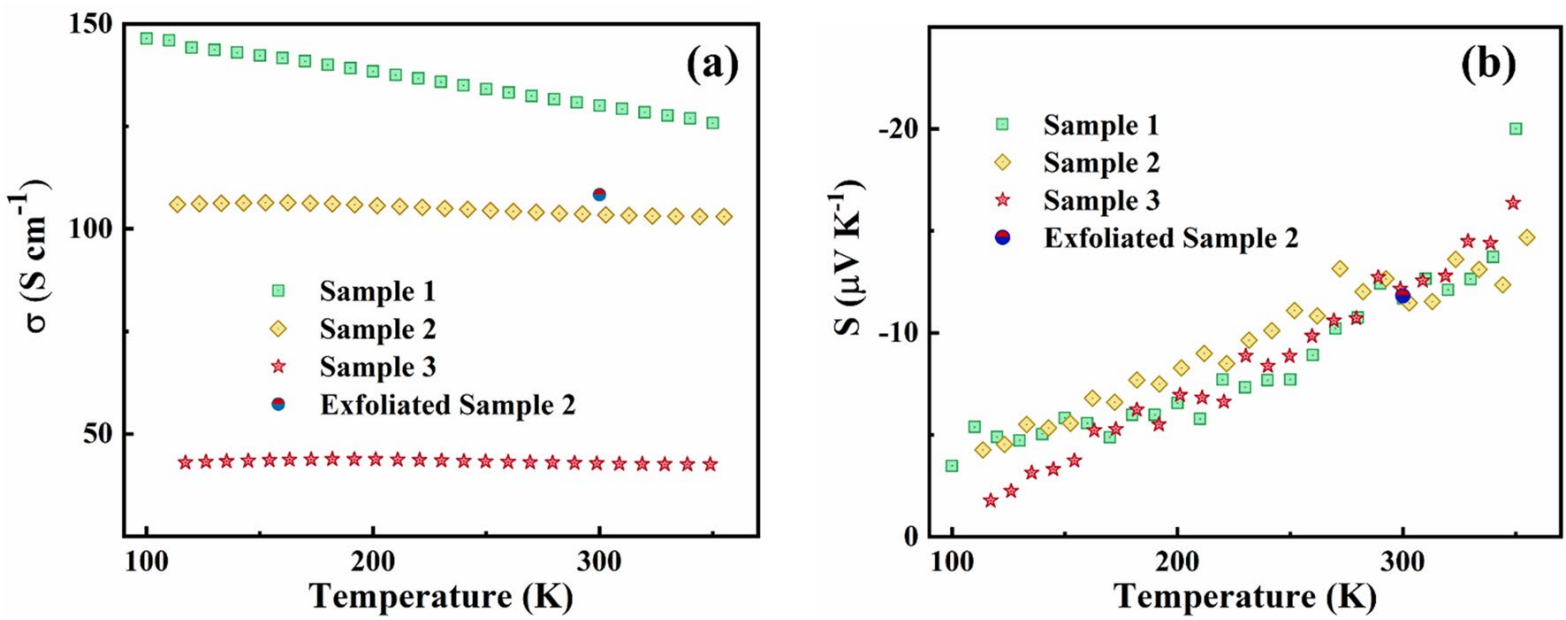
Temperature dependent electrical conductivities (**a**) and Seebeck coefficient (**b**) for different parallel combination samples (Sample 1 to Sample 3) and the exfoliated sample (Exfoliated Sample 2). The difference in electrical conductivity is a consequence of the different crystallite sizes for the samples (see Table 1).

Once the TE properties of the seed layer (*S*_*1*_ and *σ*_*1*_) have been determined, the TE properties of the parallel combination (seed layer plus electrodeposited film, see Fig. 2b) are measured (*S*_*eff*_ and *σ*_*eff*_). Figure 6a shows the electrical conductivity (*σ*_*2*_) of the electrodeposited films and Fig. 6b the Seebeck coefficient (*S*_*2*_). The first thing we observe is that the electrical conductivity, obtained by solving Eq. 1, varies between samples whilst the Seebeck coefficient is very similar. This is somewhat expected, since each sample has a different crystallite size (see Table 1). The crystallite size affects the mean free path of the electrons and hence the mobility, which in turn has an effect on electrical conductivity. The effective Seebeck coefficient, *S*_*eff*_ was measured experimentally as a function of temperature. To deduce the Seebeck coefficient for the bismuth telluride film (*S*_*2*_) from the effective Seebeck coefficient, Eq. 2 is solved. The error analysis for both S_2_ and σ_2_ is showed in detail in Supplementary Section 9. This is done for all temperatures for all samples and the result is showed in Fig. 6b. We observe that the Seebeck coefficient remains constant for the three samples, since the films were electrodeposited from the same bath and at the same deposition potential, thus expecting a similar nominal carrier concentration. It is noteworthy to mention that the Seebeck coefficient values are much lower than the typical values reported for electrodeposited films. We attribute this to a large value of carrier concentration, beyond the optimal point for Bi_2_Te_3_ (~ 5 × 10^19^ cm^−3^)^49^. According to Eq. 3, considering a scattering parameter (r) equal to − 0.5 (acoustic phonon scattering)^50,51^ and a density of states effective mass (m*) equal to m* = 0.35m_e_^52^ we obtain an expected carrier concentration (n) equal to 8.69 × 10^20^ cm^−3^ (see Supplementary Section 7). Stoltz et al. conducted similar experiment with slightly different electrodeposition conditions (they used a constant deposition potential of − 8 mV vs. SCE) and plating bath (8 mM bismuth and 10 mM telluride) and obtained very similar results^53^. Likewise, Deb et al. obtained very similar results as well, even though they modelled their seed plus film differently, attributing them to a similar hypothesis^54^. They hypothesized that the cause for this would be the uncontrollable introduction of antisite defects during the electrodeposition process. This high concentration of electrically charged defects would act as self-dopants and therefore lead to overdoping in the electrodeposited film. However, neither of the groups reported Hall measurements for these films. In order to confirm this hypothesis, we conduct Hall measurement at room temperature (see Supplementary Section 7 for further details). Due to the underneath ITO seed layer, Hall measurements cannot be conducted on the directly deposited films. Thus, we tried to mechanically remove the films from the substrate and transfer it onto insulating quartz in order to conduct Hall measurements, which could damage the films, perhaps structurally and/or electronically. Unfortunately, due to the Edisonian nature of the transfer, we could not measure all samples and only managed to successfully exfoliate Sample 2 (labelled in Fig. 6 as “Exfoliated Sample 2”). Room temperature measurements of electrical conductivity match well with the parallel combination Sample 2. Further, room temperature Hall measurements determined the carrier concentration to be 8.7 × 10^20^ cm^−3^, confirming that, indeed, the carrier concentration is very high and therefore a low Seebeck coefficient is expected. We find good agreement between the electrical conductivity of our parallel combination samples, the room temperature values of the exfoliated film and the values reported by Na et al*.*^55^. However, Na and co-workers report larger Seebeck coefficient. They attributed it to a lower carrier concentration although no Hall measurements were reported. This is explained by two factors: (a) different plating bath and different precursors (8 mM Bi powder and 10 mM Te powder), leading to a different carrier concentration and (b) different electrochemical deposition (pulsed deposition), leading to different film quality (thus having a direct impact on the crystallite size and the compactness)^55^.

The power factor (PF = S^2^σ) of these films is low, most likely due to the low Seebeck coefficient, being the maximum value 0.050 μW K^−2^ cm^−1^ at 350 K (Sample 1), as shown in Supplementary Fig. S13. These values are still far from the overdoped samples obtained by Zalar et al*.*^56^, likely due to the fact that they measured bulk samples (therefore better compactness and crystallite sizes) and we measured thin films. With respect to other electrodeposited films, reports by Trung et al*.* (PF = 0.5 μW K^−2^ cm^−1^) and Scidone et al*.* (PF = 0.60 μW K^−2^ cm^−1^), are what we should expect, since both deployed a similar electrochemical method for Bi_2_Te_3_ growth^57,58^. Na et al. used a different electrochemical technique, pulsed deposition for Bi_2_Te_3_ growth and obtained larger power factor (4.19 μW K^−2^ cm^−1^ and 4.22 μW K^−2^ cm^−1^), thanks to the improvement in the quality if the films^55^. It is noteworthy to mention that the all the aforementioned authors mechanically separated the film from the seed, thus introducing more defects in the process. This will introduce uncertainties in the measurement, as well as changing the properties of the as-deposited films.

In general, however, the power factor of electrodeposited films is lower than the power factor of other thin film deposition techniques^9^. This is due to the better quality films (compactness and morphology) that result from physically growth techniques, such as flash evaporation (PF = 18.7 μW K^−2^ cm^−1^). Bulk Bi_2_Te_3_ also show larger power factor compared to electrodeposited films. Saleemi et al*.* reported PF of 28.25 μW K^−2^ cm^−1^ from sintered powders^59^. This large PF is achieved because their spark plasma sintering procedure rendered highly densified ingots whilst preserving the nanostructure. For electrodeposited films, addition of polyvinyl alcohol to the plating bath allowed Lei et al. to improve both thickness and compactness of the films^60^. A variety of such advanced thin films can be deposited and our parallel TE measurement technique allows for direct, in-plane characterization of the Seebeck coefficient and electrical conductivity, therefore providing researchers with a way to perform such experiments in the future.

## Conclusions

Bismuth telluride thin films have been deposited onto commercially available ITO at low overpotentials. The morphology of the deposits is nodular, as expected from an electrodeposition at low over potential under mass transport regime. The films are non-stoichiometric, with the composition deviating from the ideal 40% atomic Bi – 60% atomic Te, being slightly Bi rich. The XPS analysis confirmed that most Bi is bound to Te although some appear to form an oxide Bi–O–Te. The crystallite size of the films has been calculated and the results range from ~ 21 to ~ 33 nm. A two-band model has been employed to fully understand the TE transport. The room temperature electrical conductivity of the films ranges from 40 to 100 S cm^−1^, attributed to the different crystallite sizes of the films. The Seebeck coefficient shows almost no variation for the three samples for the same temperature range, with values from − 5 μV K^−1^ to *ca.* − 20 μV K^−1^_._ It is concluded that the main difference in the herein fabricated bismuth telluride and the literature values are the carrier concentrations. Particularly, the Seebeck coefficient is lower than optimal due to a very large number of antisite defects introduced during electroplating, that act as self-dopants and increase the carrier concentration past the optimal value. This is confirmed by Hall measurements conducted on an exfoliated film (Seebeck coefficient equal to − 11.8 μV K^−1^ for a carrier concentration equal to 8.7 × 10^20^ cm^−3^). Thus, we can affirm that our method accurately measures the TE properties of electrodeposited thin films without the involuntary introduction of defects due to external forces.

## Methods

### Reagents

Bismuth telluride was electrodeposited from an aqueous electrolyte previously reported by Martin-Gonzalez et al.^14^. Briefly, 7.5 mM of Bi (elemental, Alfa Aesar, 99.999%), 10.0 mM TeO_2_ (Alfa Aesar, 99.999%) and 1 M HNO_3_ (Fisher, 69%) were dissolved in deionised water. The volume employed for electrodeposition is 15 mL. Before electrodeposition, the electrolyte was degassed for at least 20 min by gently bubbling nitrogen.

### Electrochemical techniques

Cyclic voltammetry (CV) and chronoamperometry were performed using an AutoLab Lab PGSTAT128N potentiostat/galvanostat. For CV experiments, a standard 3 electrode configuration with a Saturated Calomel Electrode (SCE, saturated KCl) reference electrode and platinum (Pt) grid counter electrode and a 1 mm diameter Pt disc working electrode (WE) were employed. For electrodeposition experiments, the reference and counter electrodes were kept the same but the WE was changed to a segment of 30 nm of indium tin oxide (ITO 80Ω/sq, composition SnO_2_:In_2_O_3_ = 1:9, Latech) on top of 1.5 mm quartz substrate of area 7 × 7 mm^2^. This 30 nm ITO on top of quartz WE will be referred to as substrate. Electrodeposition was conducted at constant potential equal to − 0.1 V vs. SCE. We kindly refer the reader to Supplementary Section 2 for further details.

### Characterization techniques

A Jeol JSM 7600F FEG-Scanning electron microscopy (SEM) was utilised to investigate the morphology of the samples. The composition of the samples was studied by Energy Dispersive X-ray spectroscopy (EDX). Spectra were collected by means of an Oxford INCA EDS detector incorporated with the INCA software, available in the Jeol JSM 7600F FEG-SEM.

XRD was conducted by means of a Bruker D8 Advance equipped with a 2D detector. The step size used for all scans was 0.02°. Phase identification was conducted using Bruker’s EVA software. Rietveld refinement was conducted using Full Prof^61^. The error of the fitting was χ^2^ = 13.5.

XPS was employed for elemental quantification as well as oxidation state identification. A Thermo Scientific Theta Probe XPS equipped with a monochromatic Al Kα X-ray (hν = 1486.7 eV) X-ray source was used for spectra acquisition. The X-ray spot was adjusted to 400 μm in diameter with an x-ray incident angle fixed to 30° with respect to normal surface. Charge compensation was achieved using low energy electron flooding. Survey scan were acquired at step = 1 eV and pass energy of 200 eV whilst the narrow scans were acquired at step = 0.1 eV and pass energy = 40 eV. Peak deconvolution and elemental quantification were conducted using the Avantage software.

## Supplementary information

Supplementary Information.

## Data Availability

Data available upon reasonable request from Jose Recatala-Gomez (j.recatala@soton.ac.uk).
