# Supplementary material for: Direct measurement of the thermoelectric properties of electrochemically deposited Bi2Te3 thin films
Source: Sci Rep. 2020 Oct 21;10:17922. doi: 10.1038/s41598-020-74887-z (PMC7578806; doi:10.1038/s41598-020-74887-z)
Supplement: Supplementary file 1 — Supplementary Information. [file 41598_2020_74887_MOESM1_ESM.docx]

**Supplementary Information**

**Direct measurement of the thermoelectric properties of electrochemically deposited Bi_2_Te_3_ thin films**

Jose Recatala-Gomez^1,2^, Pawan Kumar^1^, Ady Suwardi^1^, Anas Abutaha^1,3^, Iris Nandhakumar^2*^ and Kedar Hippalgaonkar^1,4*^

^1^ Institute of Materials Research and Engineering, Agency for Science Technology and Research, #08-03, 2 Fusionopolis Way, Innovis, Singapore 138634

^2^ Department of Chemistry, University of Southampton, University Road, Highfield, Southampton SO17 1BJ, U.K.

^3^ Qatar Environment and Energy Research Institute, Hamad Bin Khalifa University, Qatar Foundation, 34110 Doha, Qatar

^4^ School of Material Science and Engineering, Nanyang Technological University, Singapore 639798.

Corresponding author: [iris@soton.ac.uk](mailto:iris@soton.ac.uk), [kedar@ntu.edu.sg](mailto:kedar@ntu.edu.sg)

1. **Literature review on measurement of the thermoelectric properties of electrodeposited films**

This section presents the results of the survey on measurement of the TE properties of electrodeposited bismuth telluride thin film. The most widespread approach is to mechanically separate the film from the substrate (green) whilst some groups report measurement on top of the seed layer (blue).

| **Reference** | **Synthesis** | **Substrate** | **Measurement approach** |
| --- | --- | --- | --- |
| Glatz *et. al.*^1^ | Pulsed deposition | 5 nm Cr, 200 nm Au and 40nm Pt deposited on a Si wafer | Detached (method unspecified) |
| Magri *et. al.*^2^ | Galvanostatic cathodic deposition | Platinum disks | Detached (unspecified) |
| Na *et. al.*^3^ | Pulsed deposition | Stainless Steel | Detached using adhesive film substrate |
| Song *et. al.*^4^ | Potentiostatic electrodeposition | 80-nm Au layer on top of a 20-nm Ni layer deposited on a Si wafer | Detached using epoxy |
| Kang *et. al.*^5^ | Potentiostatic electrodeposition | Au layer on top of a 20-nm Ni layer deposited on a Si wafer | Detached using epoxy |
| Lei *et. al.*^6^ | Pulsed deposition | Ni foil | Detached using epoxy |
| Takahashi *et. al.*^7^ | Potentiostatic electrodeposition | Ti sheets | Detached using epoxy |
| Ma *et. al.*^8^ | Pulsed deposition | Stainless Steel | Detached using epoxy |
| Burton *et. al.*^9^ | Potentiostatic electrodeposition | 200-nm Au layer on top of a 20-nm Ti layer deposited on a Si wafer | Detached using epoxy |
| Heo *et. al.*^10^ | Potentiostatic electrodeposition | Au layer on top of a Si wafer | Detached using epoxy |
| Manzano *et. al.*^11^ | Pulsed deposition | 150 nm Pt on top of Si wafer | Detached using epoxy |
| Mizayaki *et. al.*^12^ | Potentiostatic electrodeposition | Ti sheet | Detached using epoxy |
| Wang *et. al.*^13^ | Potentiostatic electrodeposition | Platinum | Detached using epoxy |
| Calero *et. al.*^14^ | Pulsed deposition | 5nm Cr deposited on a Si wafer | Detached using epoxy |
| Yoo *et. al.*^15^ | Potentiostatic electrodeposition | Bi_2_Te_3_ growth via RF sputtering | Measured on top of the seed layer |
| Suresh *et. al.*^16^ | Potentiostatic electrodeposition | Indium Tin Oxide | Measured on top of the seed layer |
| Chen *et. al.*^17^ | Potentiostatic electrodeposition | Indium Tin Oxide | Measured on top of the seed layer |
| Cao *et. al.*^18^ | Potentiostatic electrodeposition | Bismuth telluride | Measured on top of the seed layer |
| Naylor *et. al.*^19^ | Potentiostatic electrodeposition | 100 nm Au layer on top of a segment of DVD-R disks | Measured on top of the seed layer |
| Li *et. al.* ^20^ | Potentiostatic electrodeposition | 100 nm Au layer on top of 0.1 mm of aluminium foil | Measured on top of the seed layer |
| Supplementary Table I*.* Literature review on approaches for the TE measurement of electrodeposited bismuth telluride thin films. | | | |

1. **Cyclic voltammetry and chronoamperometry**

This section elaborates on the electrochemical measurements. Cyclic Voltammetry (CV) was conducted on an electrolyte comprised by 7.5 mM Bi powder and 10 mM of TeO_2_ in 1 M HNO_3_, as described by Martin-Gonzalez *et. al.*^21^ and the result is shown in Fig. S1.

On the cathodic onset, the single reduction peak at -0.05 V vs. SCE is attributed to the deposition of bismuth telluride at the surface of the electrode, as described by Eq. S1.

3 HTeO_2_^2+^ + 2 Bi^3+^ + 18 e^-^ + 9 H^+^ → Bi_2_Te_3_ (s) + 6 H_2_O (S1)

The anodic peak at +0.40 V *vs*. SCE is assigned to the stripping of the Bi_2_Te_3_ deposited during the forward scan. The reaction that govern such process is attributed to Eq. S2. The shoulder at +0.44 V *vs*. SCE is attributed Eq. S3 and describes the oxidation of Bi to Bi^3+^.

Bi_2_Te_3_ + 6 H_2_O → 3 HTeO^2+^ + 2 Bi + 9 H^+^ + 12 e^-^ (S2)

Bi → Bi^3+^ + 3 e^-^ (S3)

In order to obtain bismuth telluride thin films, chronoamperometry is carried out at − 0.1 V vs SCE for different times (3, 5 and 7 minutes) in order to obtain films of different thicknesses.

| 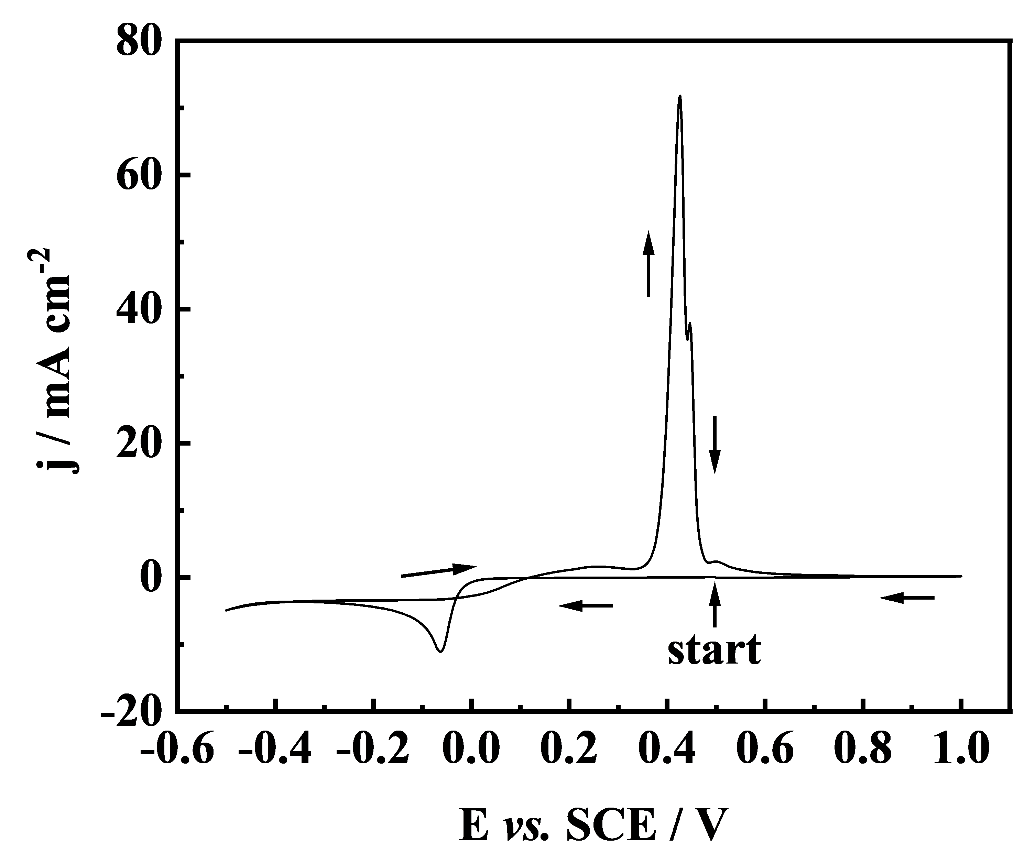 |
| --- |
| Figure S1*.* Cyclic voltammogram recorded at 20 *mV s*^-1^ in an electrolyte comprised of 7.5 *mM* Bi and 10.0 *mM* TeO_2_ dissolved in 1 *M* HNO_3_. The voltammogram started at a potential of +0.50 *V vs. SCE* where no reaction occurs and is scanned between -0.50 *V vs. SCE* and +1.00 *V vs. SCE*. The black arrows indicate the direction of the scan. The working electrode was a 1 *mm* Pt disc electrode. |

1. **Detailed XPS and XRD analysis**

This section further elaborates on the structural and chemical composition results, obtained for electrodeposited bismuth telluride. The XPS survey spectra (up to 1100 eV) showed the presence of four elements: bismuth, tellurium, carbon and oxygen, as depicted in Fig. S2. The elements were recorded for the corresponding regions of Bi 4f, Te 3d, C 1s, and O 1s. All spectra were calibrated with respect to adventitious carbon, C 1s peak at 285 eV, with an associated uncertainty of 0.2 eV.^22^ A typical survey spectrum collected for the electrodeposited bismuth telluride films is showed in Fig. S2. Fig. 1(b) shows the core-level of Te 3d spectrum. Four peaks are observed, and they are matched as follows: the peaks at 573.15 and 583.5 eV, correspond to the binding energies of spin orbit components Te 3d_5/2_ and Te 3d_3/2_ bonded to Bi, forming Bi_2_Te_3_. The other two peaks, centred at 576.2 and 586.7 eV represent the oxidized state of Te, as the binding energies match that of TeO_2_. ^23^

Fig. 1(c) shows the core-level of the Bi 4f spectrum with the well-separated spin-orbit components (splitting parameter equal to 5.3 eV) corresponding to the doublet 4f_7/2_ and 4f_5/2_, with peak positions 158.7 eV and 164 eV respectively, as previously reported.^24^ Two more peaks are shown in the core-level spectrum of Bi 4f. They correspond to the doublet 4f_7/2_ (centred at 159.2 eV) and 4f_5/2_ (centred at 164.5 eV) bound to O, therefore showing the presence of Bi_2_O_3_. This is in very good agreement with previous reports. ^23,24^ The ratio atomic Bi to atomic Te was also calculated from the areas of the Bi 4f_7/2_ and the area of the Te 3d_5/2_ and the results are summarised in Table I. If the elemental analysis showed is compared with the elemental analysis as per determined by EDX, a composition mismatch is observed between the techniques, attributed to the different sampling depth between the techniques. XPS is a surface sensitive technique with sampling depth of 10 nm whilst EDX has a sampling depth in the range of 3 μm.^25,26^

| 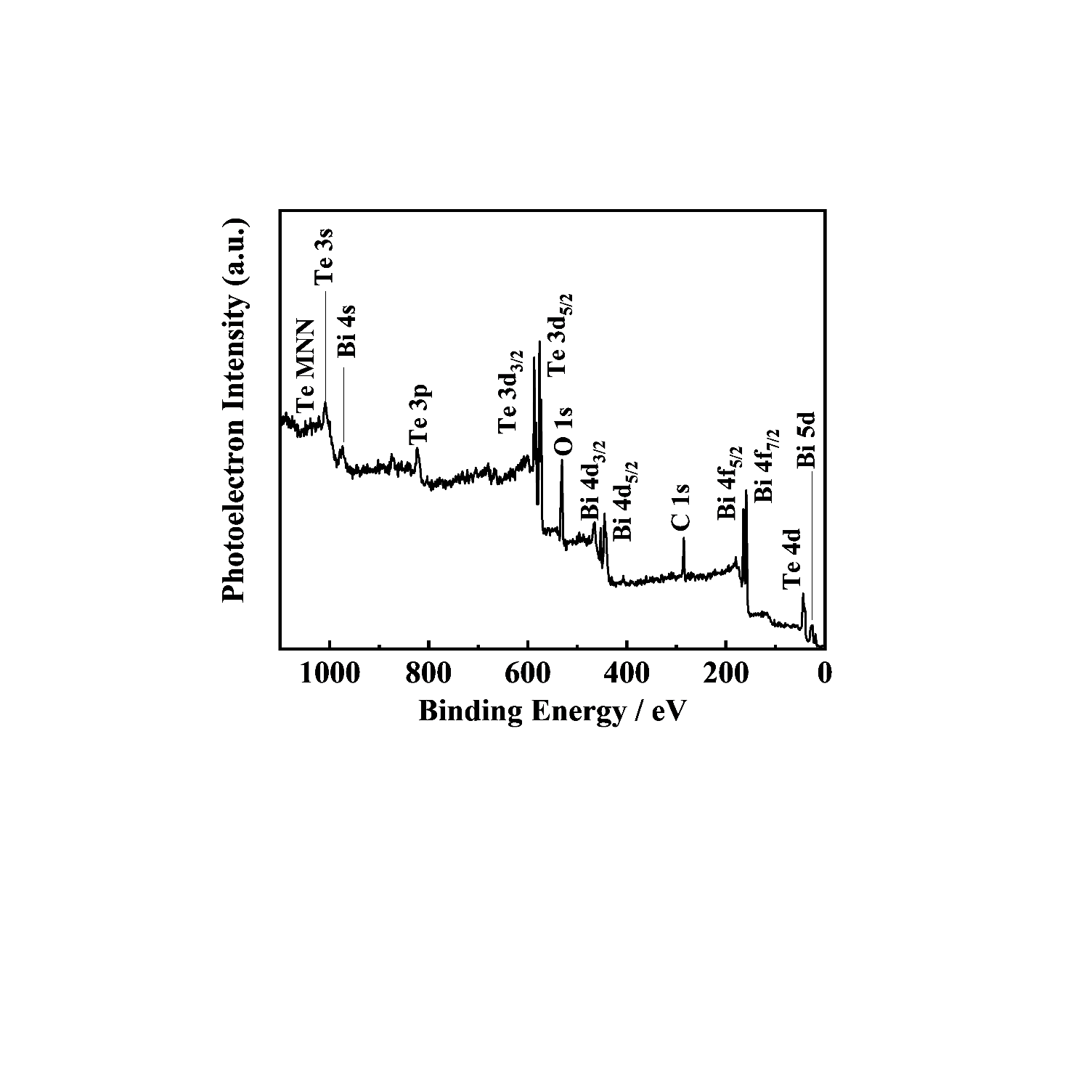 |
| --- |
| Figure S2. Typical survey spectrum collected for the electrodeposited films. |

In order to investigate the crystal structure of the electrodeposited films, two-dimensional diffraction image frames were collected with frame centres set to 30, 50 and 70 degrees in 2Ɵ, from right to left, and then merged. The corresponding 1D XRD spectrum was obtained by integrating the frames from 2Ɵ = 20° to 2Ɵ = 80°. The results are summarised in Fig. 1(d) in the manuscript. The crystallite size (D_XRD_) of the films was calculated using the Scherrer Equation (D_XRD_ = Kλ/Bcosθ, where K is the Scherrer constant, equal to 0.94, λ is the wavelength of x-ray source, λ = 0.15406 nm, B is the full width at half maximum in radians and cosθ is the cosinus of the Bragg angle, θ)^27^ based on the XRD peak broadening of the (015) peak, determined using the Fityk® software.^28^

1. **Detailed analysis of the TE measurement of ITO**

Herein, we discuss about thermoelectric measurement in details. Fig. 2 in the main manuscript shows a schematic representation of the fabrication of the device. The fabrication starts with cleaning the substrate by sonication in acetone and isopropyl alcohol (IPA) for 5 minutes each. Thereafter, a segment of 1 x 7 mm of Kapton tape is used to mask a part of ITO in the centre of the device (Fig. 2(a), step 1). The second step in the fabrication is the wet etching of the ITO by immersion of the masked substrate in 10 mL of HCl 6M (36% purity, electronic grade, Megachem Limited) for 15 minutes. As a result, the substrate is now all quartz except the stripe of ITO (prevented from etching by the Kapton tape), showed in Fig. 2(a), after step 2 (light blue stripe in the middle represents the ITO that has been left untouched thanks to the mask. The rest of the substrate is quartz). The third step is the electrodeposition of the bismuth telluride, which will be deposited only on top of the masked ITO (grey stripe in Fig. 2(a), after step 3).

The temperature dependent resistances of both thermometers were measured, as depicted in Fig. 4(a). As previously reported, the resistance drops with the temperature increase for both thermometers. ^29^ The curve was fitted to a straight line with equation R(T) = R_0_ + AT (black lines in Fig. 4(a)). The fitting parameter A for the hot (top) and cold (bottom) thermometers is 0.068 ± 2.440 10^-4^ Ω K^-1^ and 0.068 ± 1.957 10^-4^ Ω K^-1^_._ The Pearson’s R-Square for both linear fits were almost unity (≈0.999). The hot thermometer (top) shows more resistance change due to the higher local temperature, in line with the literature. ^29^ Fig. S3(a) shows the resistance of both thermometers as a function of the current at room temperature. The parabolic dependence between the resistance and the current follows a Joule heating dependence (P ∝ I^2^R). Therefore, a parabolic fit of equation R(I) = CI^2^ + D is performed (solid red and blue curves in Fig. S3(a)) for all temperatures. The room temperature values of the fitting parameters C and D were found to be 837.24 ± 65.22 Ω/A^-2^ and 33.30 ± 0.05 Ω for the hot thermometer and 1039.89 ± 55.19 Ω/A^-2^ and 32.17 ± 0.04 Ω for the cold thermometer. With the appropriate combination of the slopes of the thermometers (Fig. 4(a)) and fitting parameters of the parabolic curves in Fig. S3(a), the change in resistance and thus the temperatures at the hot thermometer (T_hot_) and the cold thermometer (T_cold_) were obtained.

The Seebeck coefficient of the sample is obtained by measuring the open circuit voltage (V_oc_), according to Eq. S4:

$S_{sample}= -\frac{V_{oc}}{\Delta T}+ S_{Aluminium}$ (S4)

where S is the Seebeck coefficient, ΔT is the difference between the hot and cold temperature (ΔT = T_hot_ - T_cold_) and S_Aluminium_ is the Seebeck coefficient of the aluminium. This term has to be included because the measurement device has been connected with aluminium wires.^29^

Fig. S3(b) shows the V_oc_ dependence with the heater current at room temperature. It also rises parabolically, as the heating is generated by Joule heating. The same parabolic fitting is performed for all temperatures. For room temperature, the fitting parameters are determined as C = -18504.60 ± 3040.47 μV A^-2^ and D = -755.19 ± 2.43 μV. Finally, the Seebeck coefficient for the sample (S_sample_) is obtained by calculating the slope of the V_oc_ vs. ΔT (Fig. 4(b), linear fit corresponds to solid red line) and adding the corresponding Seebeck coefficient for the aluminium at that temperature.

| 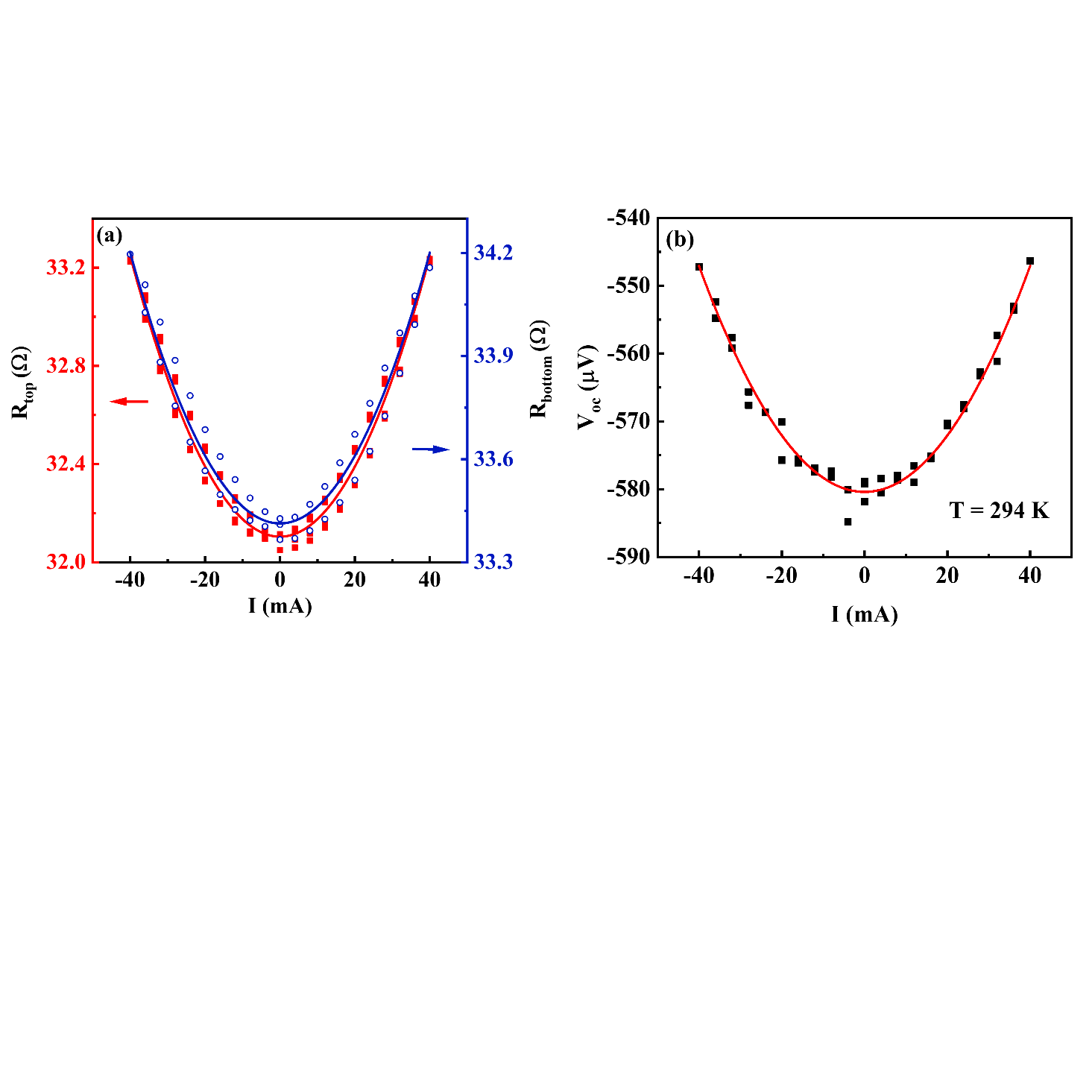 |
| --- |
| Figure S3. (a) Change in resistance as a function of heater current for hot and cold thermometers at room temperature. The red and blue lines correspond to the parabolic fit R(I) = CI2 + D for the hot and cold thermometers, respectively. (b) Open circuit voltage dependence with heater current. |

1. **Hall effect measurement the ITO film**

To further understand the transport properties of the seed layer, temperature dependent Hall measurements have been carried out with a Quantum Design® Physical Property Measurement System (PPMS). Fig. S4(a) shows the dependence of the Hall resistance with the applied magnetic field (H). The Hall resistance increases monotonically with the magnetic field. The Hall resistance relates to the carrier concentration (in the degenerate regime) as per Eq. S5, where V_H_ is the Hall voltage, I is the current, H is the applied magnetic field, t is the thickness of the seed layer (30 nm), n is the carrier concentration and q is the elemental charge (1.602 10^-19^ C). Thus, if a linear fit is performed and the slope is obtained, the carrier concentration can be calculated.

$R_{H}=\frac{V_{H} t}{I H}=\frac{-1}{nqt}$ (S5)

Fig S4(b) shows the carrier concentration as a function of the temperature. At all temperatures, the carrier concentration has values in the order of 10^21^ cm^-3^, with no abrupt changes. These carrier concentration values indicate that ITO conduction is of metallic nature.^30^ This is in very good agreement with the literature. ^31^ Lin *et. al.* fabricated ITO by means of RF sputtering, reporting crystallite sizes in between 17-25 nm and carrier concentrations of 2.6-5.6x10^20^ cm^-3^, with no mobility reported. ^32^ Li *et. al.* studied the thermoelectric properties of commercially available ITO films fabricated by means of RF sputtering. They calculated their carrier concentration to be 9.8x10^20^ cm^-3^ (no crystallite size or mobility was reported). ^33^ Kytin *et. al.* deposited ITO films at room temperature and 230 ^o^C by means of DC magnetron sputtering (target with composition 90 wt % In_2_O_3_ and 10 wt % SnO_2_), with no crystallite size reported. To their knowledge, amorphous films are expected from deposition at room temperature. Regarding the films deposited at 230^o^C, previous studies show that ITO grown on silicon shows crystallite sizes ranging from 15-20 nm. This of course may differ from the real result since in their report amorphous glass as substrate was used. The corresponding carrier concentrations at 4.2 K for these films are 6.4x10^20^ cm^-3^ and 4.9x10^20^ cm^-3^, with mobility values of 15 cm^2^ V^-1^ s^-1^ and 37 cm^2^ V^-1^ s^-1^. The value of effective mass employed by these authors was m^*^ = 0.35m_e_.^34^ Liu *et. al.* fabricated ITO thin films on glass by means of electron beam evaporation, from pellets with composition 95 wt % In_2_O_3_ and 5 wt % SnO_2_. ^30^ The authors did not report crystallite sizes, but the carrier concentrations were determined to be 2.78x10^21^ cm^-3^ and 3.42x10^21^ cm^-3^.

| 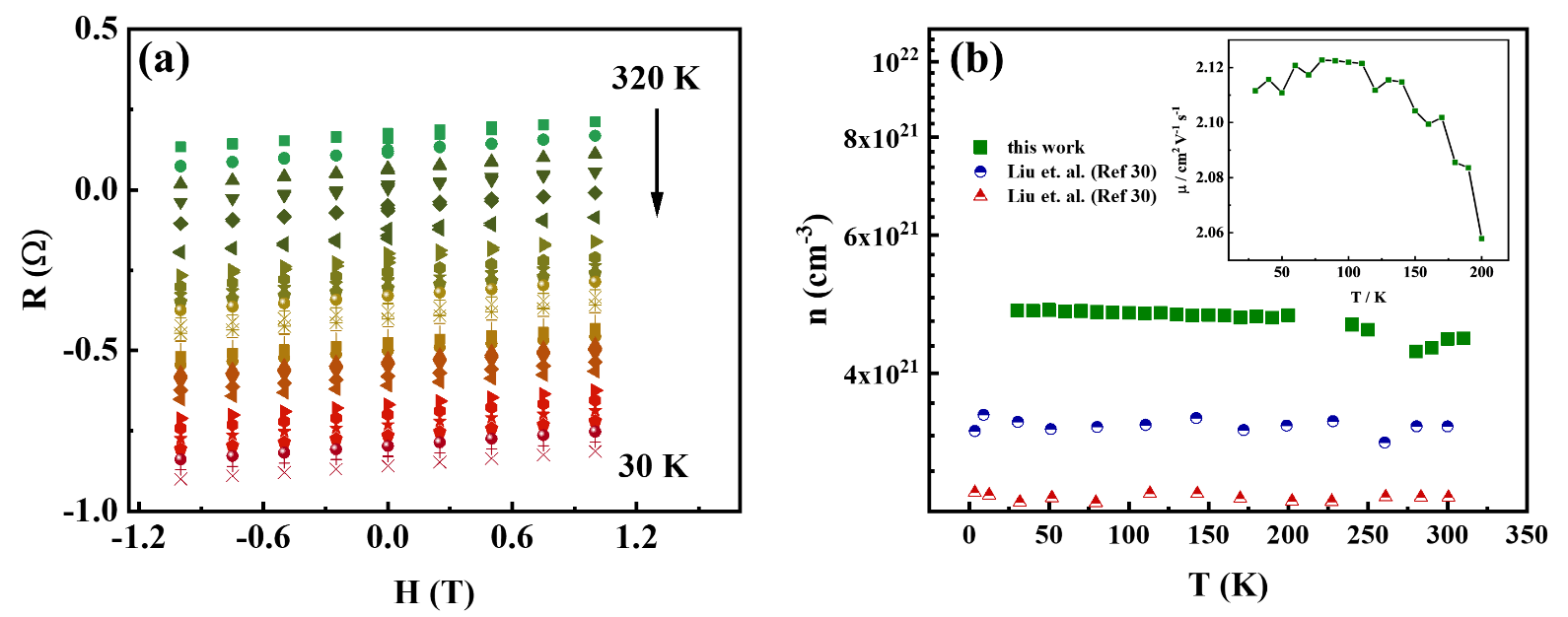 |
| --- |
| Figure S4*.* (a) Temperature dependent resistance vs magnetic field and (b) carrier concentration as a function of the temperature. The experimental data is compared with previously reported data. Inset in (b): temperature dependence of mobility for the seed layer. The mobility drops as the temperature is increased. |

The Seebeck coefficient can be calculated by means of Eq. 3 (see main text). Here, the value of effective mass is m = 0.30m_e_^35^ and r is equal to -0.5 (acoustic phonon scattering). ^36^ Once the Seebeck values are obtained, a Pisarenko plot can be drawn for ITO and the result is shown in Fig. S5. As expected for carrier concentrations in the degenerate (metallic) regime, the data is located in the plateau of the Pisarenko plot, indicating that the Seebeck will not change with doping, typical behaviour in metals and heavily doped semiconductors. This demonstrates that our measurement is coherent and in line with the previously reported values. ^35^

| 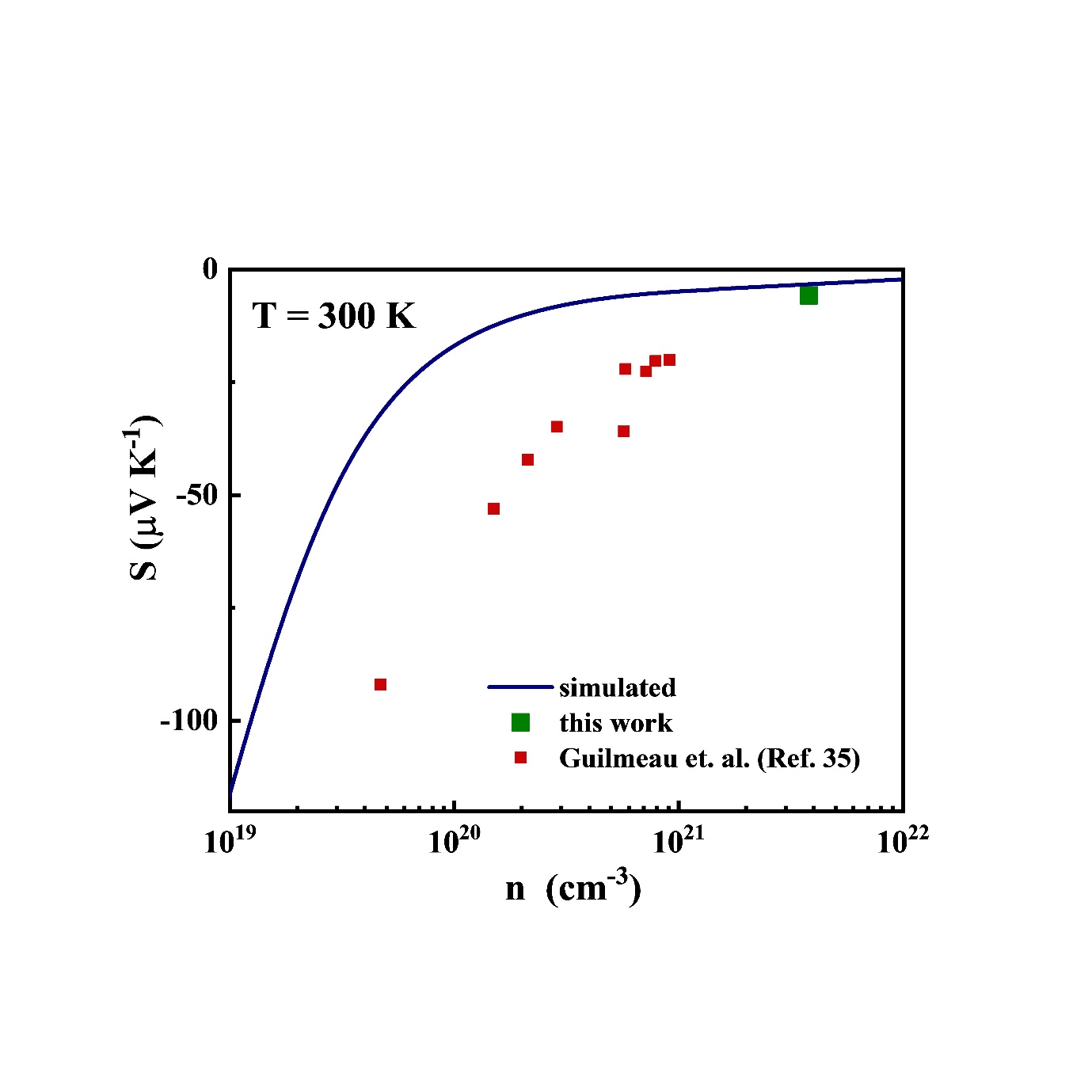 |
| --- |
| Figure S5. Pisarenko plot (S vs. n) for T = 300 K for the experimental data compared with the results from Guilmeau *et. al.*^35^ The simulated curve has been calculated by solving Eq. 3, taking m^*^ = 0.30m_e_ for ITO. |

1. **TE data for electrodeposited bismuth telluride films**

The TE data for bismuth telluride (Fig. S6 to Fig. S8), at room temperature is depicted in this section. The reader must note that the analysis is analogous as the analysis described for the TE measurement of ITO. Likewise, this same analysis is done for all temperatures and as a result, the temperature dependent electrical conductivity and Seebeck coefficient for the electrodeposited Bi_2_Te_3_ is obtained, as depicted in Fig. 6(a) and (b), respectively.

| 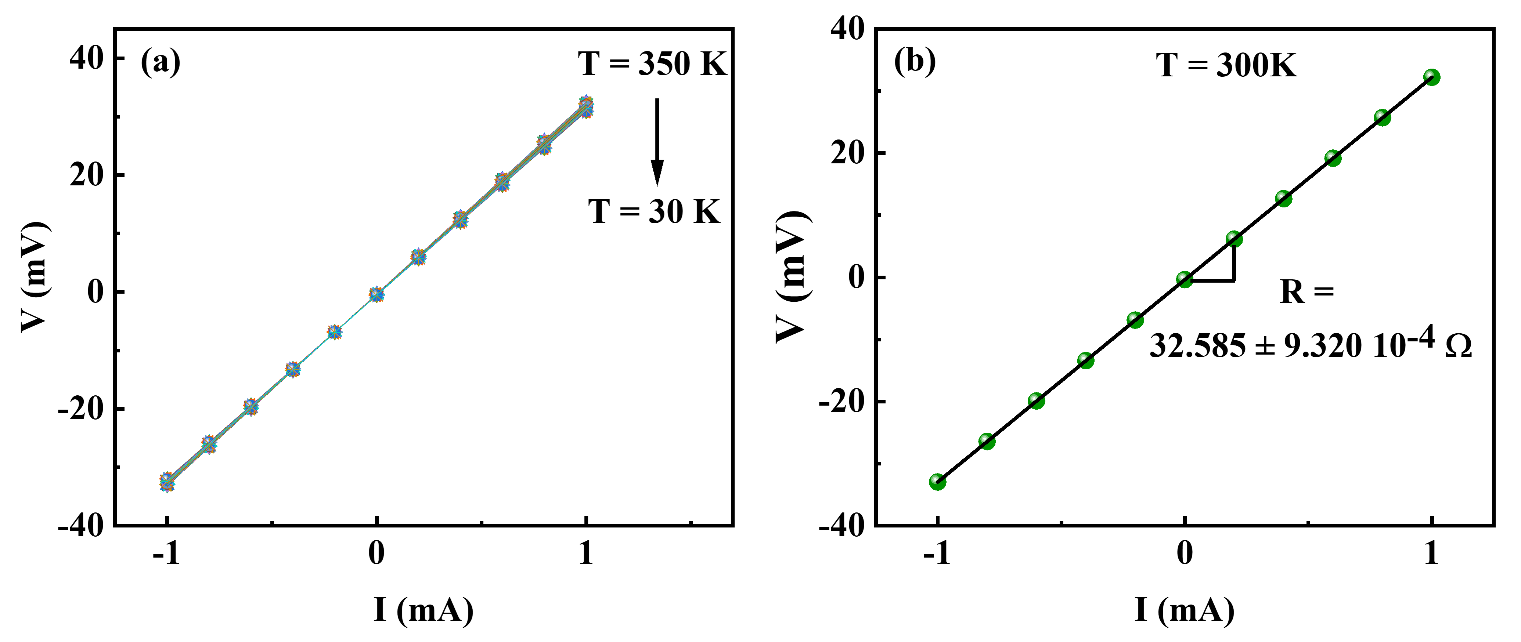 |
| --- |
| Figure S6. (a) Four-probe I-V curves for different temperatures. (b) curve for T =300K. The black line indicates a linear fit in order to calculate the resistance (R = 32.585 ± 9.320 10^-4^ Ω). |

| 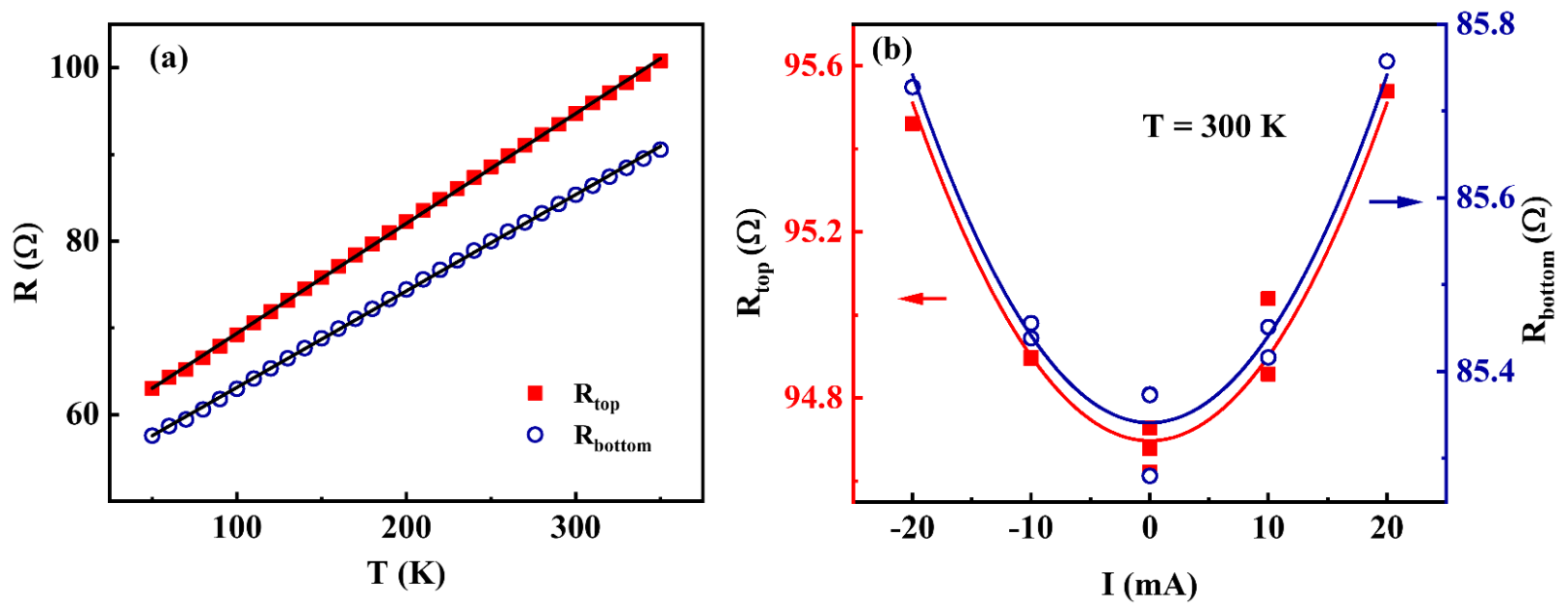 |  |
| --- | --- |
| Figure S7*.* (a) Change in resistance of both thermometers, *R_top_* is the resistance of hot thermometer and *R_bottom_* is the resistance of cold thermometer, as a function of temperature for the parallel combination ITO + Bi_2_Te_3_. The black line corresponds to the linear fit R(T) = R0 + AT. (b) Change in resistance as a function of heater current for hot and cold thermometers at 300K. The red and blue lines correspond to the parabolic fit R(I) = aI^2^ + b for the hot and cold thermometers, respectively. | |

| 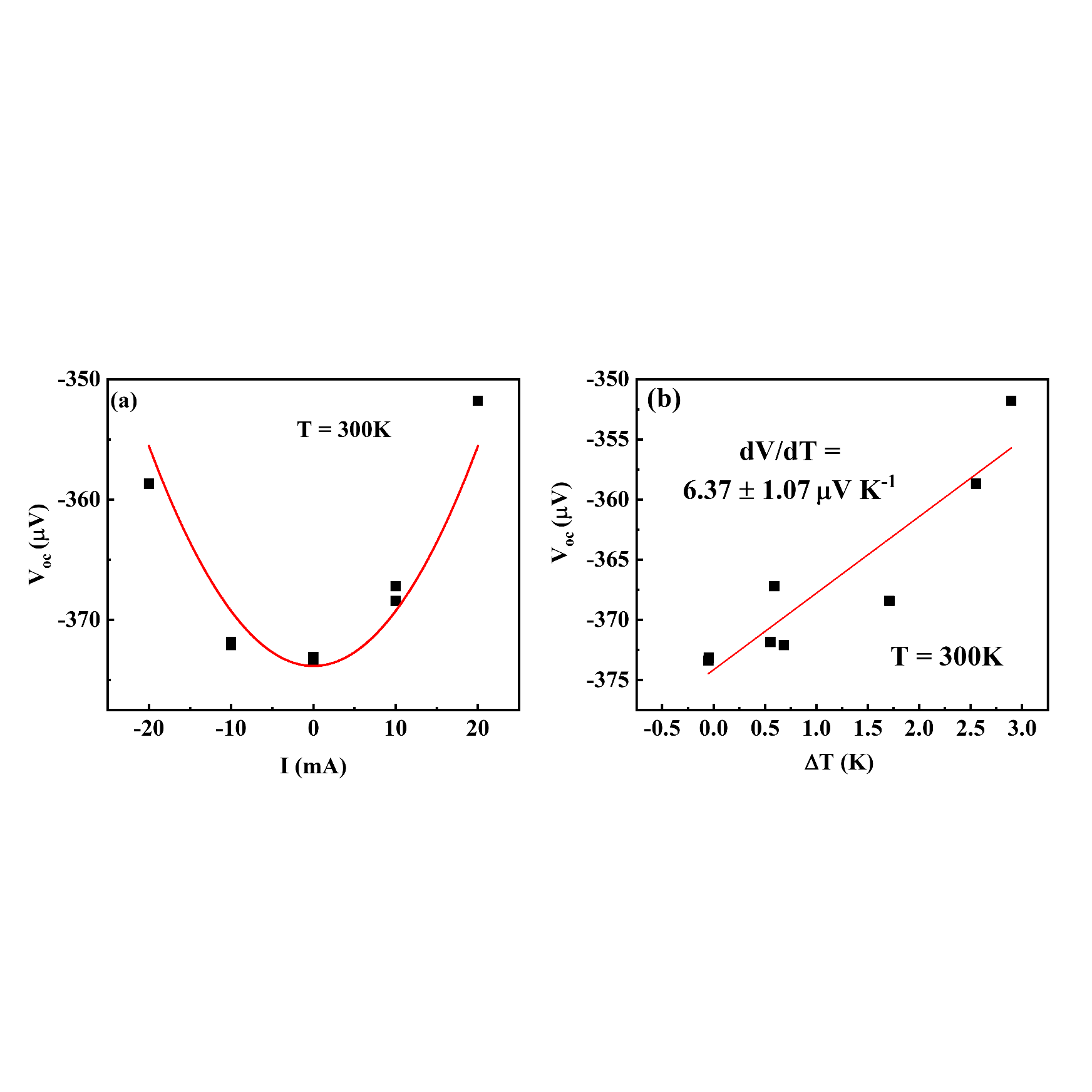 |  |
| --- | --- |
| Figure S8*.* (a) Open circuit voltage dependence with heater current. The positive curvature of V_oc_ indicates that the material is *n*-type. (b) Open circuit voltage dependence with the temperature difference. The red line corresponds to a linear fit in order to obtain the Seebeck coefficient for the parallel combination of ITO and Bi_2_Te_3_. | |

1. **Hall effect measurement of exfoliated Bi_2_Te_3_ film**

We hypothesized that the low values of Seebeck coefficient obtained for our films are due to a very high carrier concentration. Thus, a representative film is electrodeposited from the same bath and same conditions as Sample 2, described in Methods (see main text) and Supplementary Section 2. Thereafter, the film is mechanically separated (RS PRO epoxy was used) and transferred on a 7 x 7 mm quartz segment (thereby named *“Exfoliated Sample 2”*) using the same approach as followed in the corresponding references in Supplementary Section 1. The contacts are evaporated as described in the main manuscript. Hall measurements are conducted at room temperature in order to find out the carrier concentration and the result is showed in Supplementary Figure S9. We observe a linear correlation between Hall resistance and applied magnetic field, with hysteresis to some extent. The hysteresis can be attributed to the lack of device integrity that we expect in the exfoliated samples, and the inability to form good electrical contact during evaporation of the metal, highlighting that exfoliation and removal of electrodeposited samples is not a good strategy. From the Hall resistance *vs.* applied magnetic field dispersion and using the Supplementary Equation S5, the room temperature carrier concentration is calculated to be 8.7 x 10^20^ cm^-3^. This value is much higher than the values reported by Zalar for overdoped Bi_2_Te_3_ and therefore lower Seebeck coefficient should be expected.^37^

| **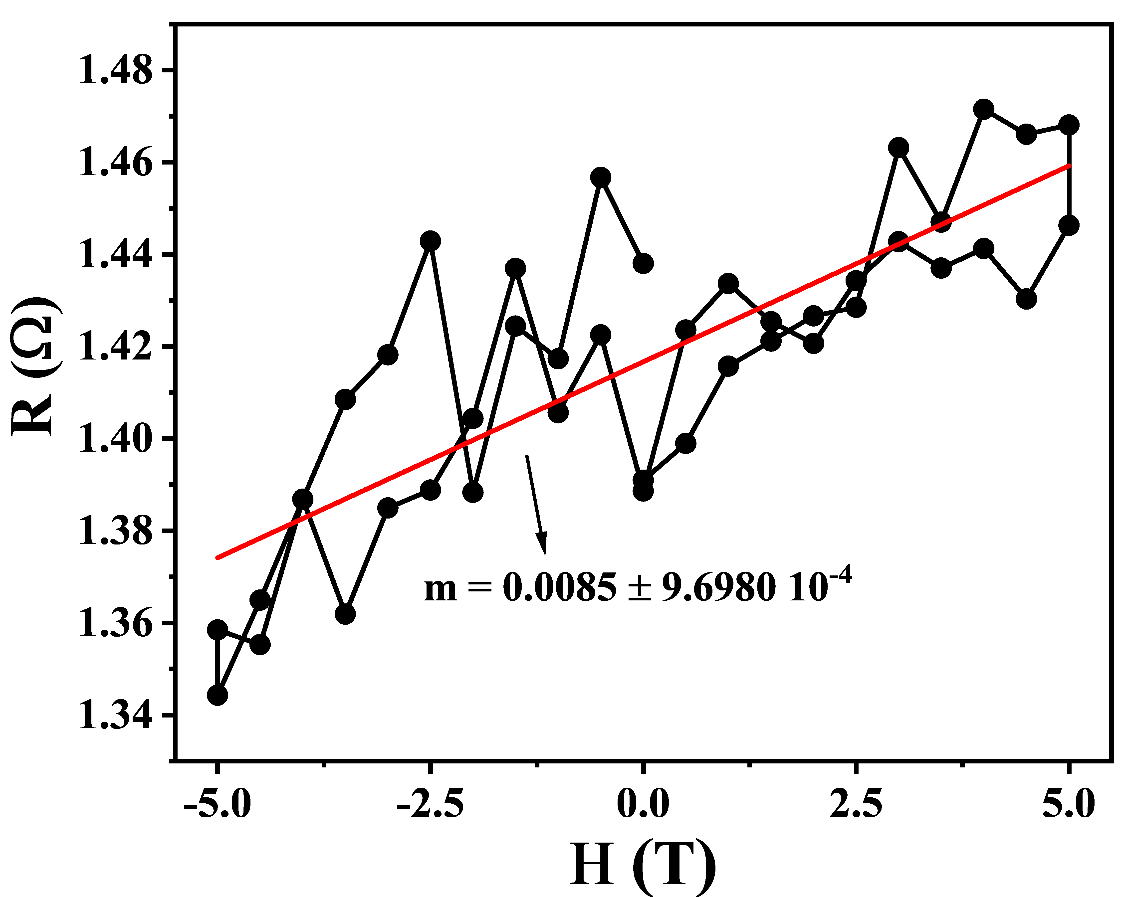** |
| --- |
| Figure S9. Room temperature Hall resistance (R_H_) vs magnetic field (H) for a representative electrodeposited bismuth telluride thin film. The film was removed from the conducting seed layer and transferred to quartz using epoxy. The red line corresponds to a linear fit performed to calculate the slope of the R_H_ vs. H field for further calculations and the value of the slope (m) is taken for calculation of the carrier concentration.  In the main text we mention that the Mott formula captures the trend but does not give accurate numbers and therefore the full BTE is used. Figure S10 shows the relationship between S and n, calculated using the full BTE (Eq. 4) and its approximation, the Mott formula.^38^ For a fixed value of Seebeck coefficient, S = -11.8 µV K^-1^ (as experimentally determined, see Fig. 6(b), Exfoliated Sample 2) the carrier concentration given by the full BTE is n = 8.7 x 10^20^ cm^-3,^ in good agreement with the value determined by Hall measurement. On the other hand, the Mott equation predicts a carrier concentration of n = 4.4 x 10^21^ cm^-3^. This disparity is due to the limitations of the Mott formula and therefore care must be taken when using it.   \| 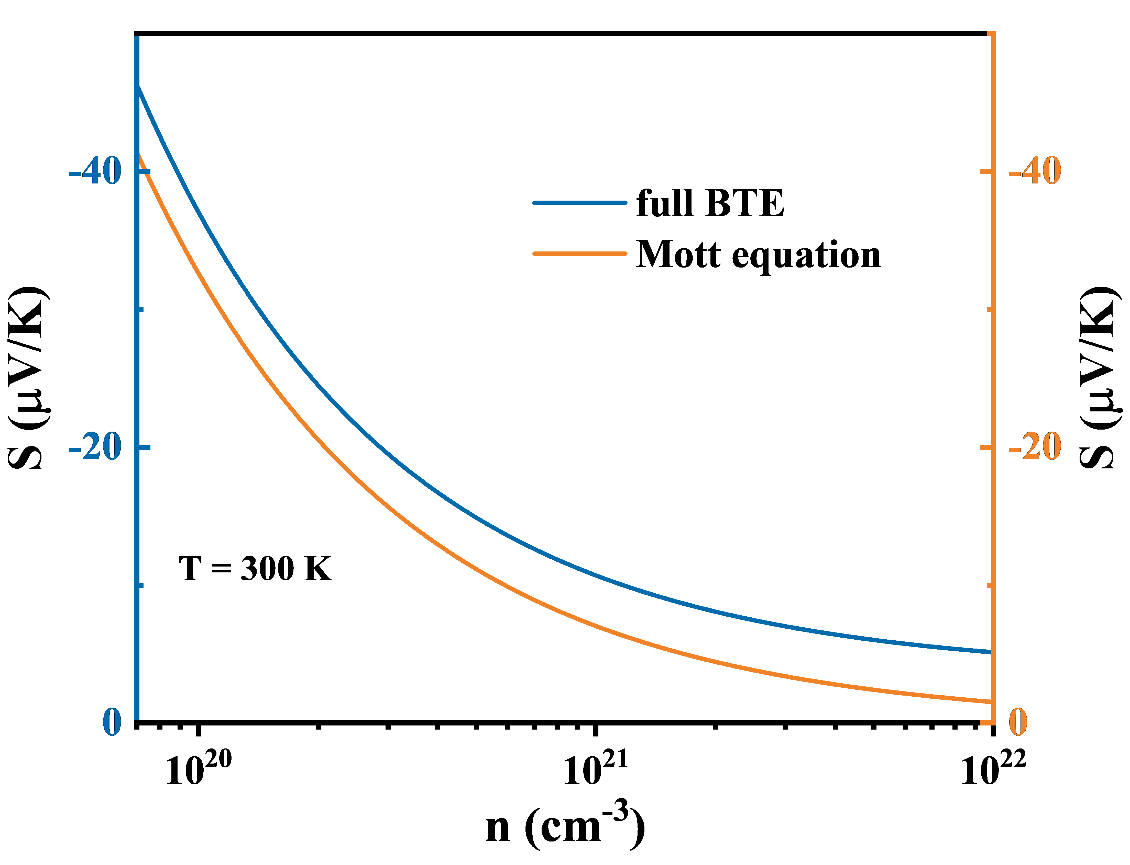 \| \| --- \| \| Figure S10. Theoretical Pisarenko plot, calculated by the full Boltzmann Transport Equation (Eq. 3 in the main text). \| |

1. **Experimental data for traditional electrodeposition of bismuth telluride.**

Traditional electrodeposition of bismuth telluride employs noble metals, mostly gold as seed layer. This is because gold has two qualities that electrochemists value in a seed layer: is it highly conducting (ensuring a good electrical contact) and it does not oxidise (so no interference from native oxide layers). In order to show that gold is not a good seed layer for us and measurements on top of gold are not accurate, we fabricated a working electrode comprised of 100 nm of Au (on top of a 10 nm Ti seed layer) by means of a Denton Explorer 14® e-beam thermal evaporator. We then electrodeposited bismuth telluride in the same way as described in the Methods section of the main text and in Supplementary Section 2. We first measured the electrical properties of the seed layer and then proceeded to measure the properties of the parallel combination, as explained in the manuscript. The results that were obtained are summarised in Supplementary Figure S11. The parallel combination shows resistances in the same order as the gold seed layer. Hence, the measurement is short-circuited. This indicated that gold is not a good seed layer according to our model.

| *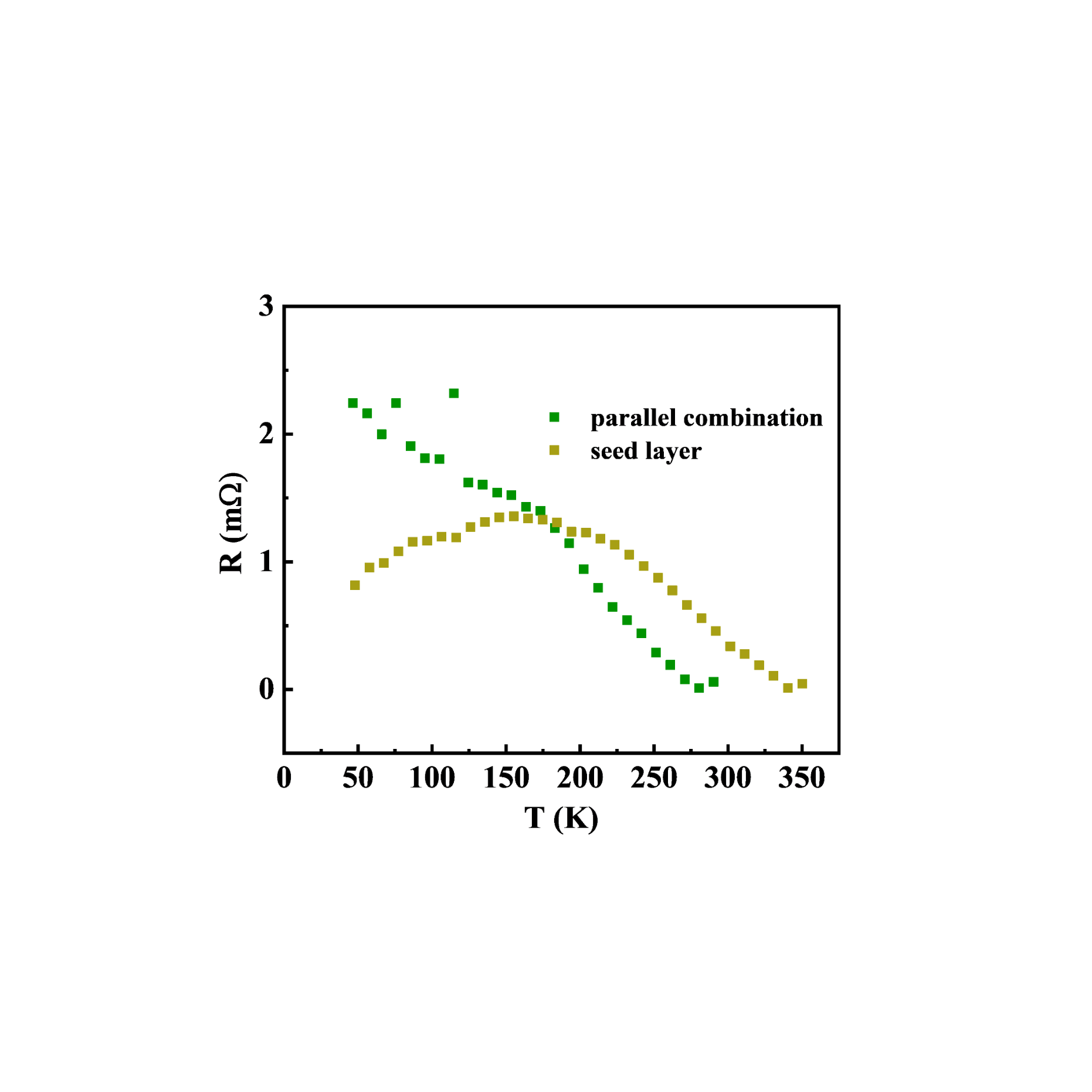* |
| --- |
| Figure S11*.* Resistance as a function of temperature for a frequently employed seed layer (100 nm of Au on top of 10 nm of Ti adhesion layer) and an electrodeposited Bi_2_Te_3_ thin film. |

1. **Error analysis**

The calculation of the error in Seebeck coefficient and electrical conductivity is analogous to the error analysis carried out by Kumar *et. al.*^29^ The reader is kindly referred to this reference for details about the analysis. First let us consider the error in electrical resistivity (ρ = 1/σ, where ρ is the electrical resistivity and σ the electrical conductivity). The error in resistivity is calculated according to Supplementary Equation S6.

$\frac{\delta\rho}{\rho}= \sqrt{\left( \frac{\delta(R)}{R} \right)^{2}+ \left( \frac{\delta(w)}{w} \right)^{2}+\left( \frac{\delta(t)}{t} \right)^{2}+ \left( \frac{\delta(L)}{L} \right)^{2}}$ (S6)

where R is the 4-probe resistance, w is the width of the film, t is the thickness of the film, L is the channel length (distance between V^+^ and V^-^ electrodes) and δ() indicated the error of the parameter contained in the brackets. In this work, L is fixed to 1 mm (see Kumar *et. al.* for more details).^29^ The resistance is calculated by taking the slope of the I-V curves. As shown in Figures 3(a) of the main manuscript and S6(a) and (b), the I-V curves are linear, with R^2^ ≈ 1, so the error in R is negligible. The geometric parameters of the film, t and w, are measured by means of a KLA-Tencor profilometer that has a minimum step height (and thus error, δt) of ± 1 nm and a scan resolution of 0.2 µm, with a scan rate of maximum value 10 µm. Henceforth, the errors in channel length and width are also negligible due to their larger magnitude with respect to their error.^29^ Therefore, the error in resistivity is given by Supplementary Equation (S7):

$\frac{\delta\rho}{\rho}= \frac{\delta t}{t}$ (S7)

Therefore, the thicker the film the less the error in the measurement. Thus, the less error in resistivity and in turn in conductivity. The thinnest film with electrical properties measured in this work is the ITO seed layer (30 nm). According to S7, the error in this measurement is 3.33 %. The electrodeposited Bi_2_Te_3_ thin films have thicknesses larger than 1 µm (see Table 1 in the main text). Therefore, the error in these measurements is considered negligible.

The error in Seebeck comes mostly from the error in measuring the temperature difference.^29^ Fig. S12 shows corresponding change in temperature with the error bar as a function of heater current for both hot (ΔT_h_) and cold (ΔT_c_) thermometers. The error is highlighted by means of the inset in Fig. S12.

| 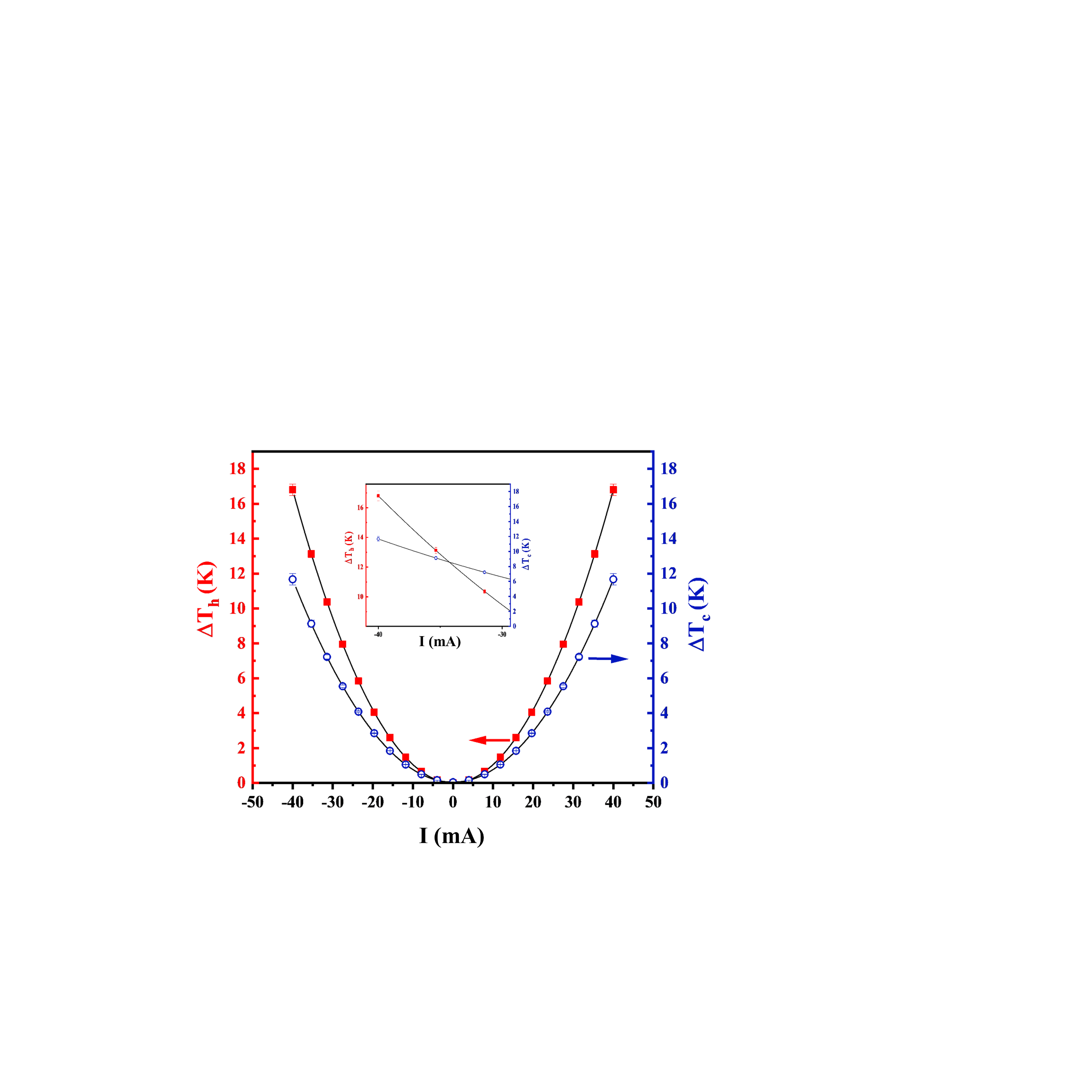 |
| --- |
| Figure S12. Change in temperature with heater current for the hot (ΔT_h_) and cold (ΔT_c_) thermometer. The inset is meant to give a better insight into the small error of the measurement. |

Regarding the error in the measurement of the Seebeck coefficient of the electrodeposited bismuth telluride thin films (S_2_ in Eq. (2) of the main manuscript):

$S_{2}= S_{eff}+\frac{S_{eff} \sigma_{1} t_{1}}{\sigma_{2} t_{2}}- \frac{S_{1} \sigma_{1} t_{1}}{\sigma_{2} t_{2}}$ (S8)

The error in S_2_ will be given by:

$\delta S_{2}= \sqrt{\left( \delta S_{eff} \right)^{2}+ \left( \delta(\frac{S_{eff} \sigma_{1} t_{1}}{\sigma_{2} t_{2}}) \right)^{2}+\left( \delta(\frac{S_{1} \sigma_{1} t_{1}}{\sigma_{2} t_{2}}) \right)^{2}}$ (S9)

If the individual components are expanded to calculate their errors:

$\delta S_{eff}$: As showed in Fig. S12, the error in Seebeck measurement is equivalent to the error in the measurement in ΔT and is very small.

$\delta\left( \frac{S_{eff} \sigma_{1} t_{1}}{\sigma_{2} t_{2}} \right)= \frac{S_{eff} \sigma_{1} t_{1}}{\sigma_{2} t_{2}} \sqrt{\left( \frac{\delta S_{eff}}{S_{eff}} \right)^{2}+ \left( \frac{\delta\sigma_{1}}{\sigma_{1}} \right)^{2}+\left( \frac{\delta t_{1}}{t_{1}} \right)^{2}+\left( \frac{\delta\sigma_{2}}{\sigma_{2}} \right)^{2}+\left( \frac{\delta t_{2}}{t_{2}} \right)^{2}}$ (S10)

$\delta\left( \frac{S_{1} \sigma_{1} t_{1}}{\sigma_{2} t_{2}} \right)= \frac{S_{1} \sigma_{1} t_{1}}{\sigma_{2} t_{2}} \sqrt{\left( \frac{\delta S_{1}}{S_{1}} \right)^{2}+ \left( \frac{\delta\sigma_{1}}{\sigma_{1}} \right)^{2}+\left( \frac{\delta t_{1}}{t_{1}} \right)^{2}+\left( \frac{\delta\sigma_{2}}{\sigma_{2}} \right)^{2}+\left( \frac{\delta t_{2}}{t_{2}} \right)^{2}}$ (S11)

Supplementary Eq. (6) and (7) show the derivation for the errors in conductivity and thickness. For example, in Sample 2 (t = 2.5 µm) the error in the resistivity measurement is 1.20 %. For this sample at room temperature, the error in S_2_ is calculated to be 12.06 %.

1. **Temperature dependent power factor for electrodeposited and exfoliated bismuth telluride thin films**

| 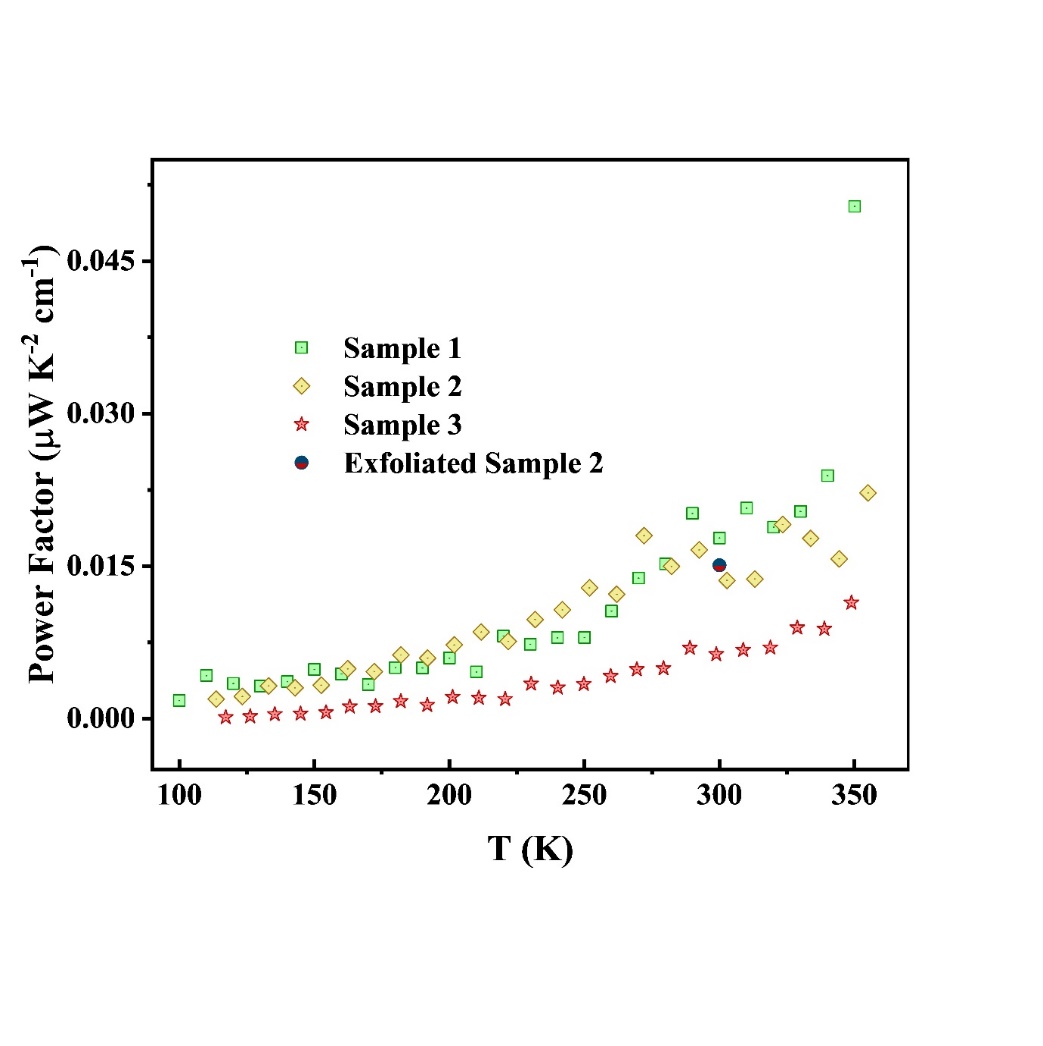 |
| --- |
| Figure S13. Temperature dependent power factor (S^2^σ) for the electrodeposited bismuth telluride thin films (Sample 1 to Sample 3) and the exfoliated sample (Exfoliated Sample 2). |

**Supplementary References**

1. Glatz, W., Durrer, L., Schwyter, E. & Hierold, C. Novel mixed method for the electrochemical deposition of thick layers of Bi2+xTe3-xwith controlled stoichiometry. *Electrochim. Acta* **54**, 755–762 (2008).

2. Magri, P., Boulanger, C. & Lecuire, J.-M. Synthesis, properties and performances of electrodeposited bismuth telluride films. *J. Mater. Chem.* **6**, 773 (1996).

3. Na, J., Kim, Y., Park, T., Park, C. & Kim, E. Preparation of Bismuth Telluride Films with High Thermoelectric Power Factor. *ACS Appl. Mater. Interfaces* **8**, 32392–32400 (2016).

4. Song, Y., Yoo, I-J., Heo, N-R., Lim, D. C., Lee, D., Lee, J. Y., Lee, K. H., Kim, K.-H., Lim, J.-H. Electrodeposition of thermoelectric Bi2Te3 thin films with added surfactant. *Curr. Appl. Phys.* **15**, 261–264 (2015).

5. Kang, W. S., Li, W. J., Chou, W. C., Tseng, M. F. & Lin, C. S. Microstructure and thermoelectric properties of Bi2Te3electrodeposits plated in nitric and hydrochloric acid baths. *Thin Solid Films* **623**, 90–97 (2017).

6. Lei, C., Burton, M. R. & Nandhakumar, I. S. Facile production of thermoelectric bismuth telluride thick films in the presence of polyvinyl alcohol. *Phys. Chem. Chem. Phys.* **18**, 14164–14167 (2016).

7. Takashiri, M., Makioka, T. & Yamamuro, H. Promotion of crystal growth in as-grown Bi2Te3 electrodeposited films without micro-pores using sputtered Bi2Te3 seed layers deposited on a glass substrate. *J. Alloys Compd.* **764**, 802–808 (2018).

8. Ma, Y., Ahlberg, E., Sun, Y., Iversen, B. B. & Palmqvist, A. E. C. Thermoelectric properties of thin films of bismuth telluride electrochemically deposited on stainless steel substrates. *Electrochim. Acta* **56**, 4216–4223 (2011).

9. Burton, M. R., Richardson, S. J., Staniec, P. A., Terrill, N. J., Elliott, J. M., Squires, A. M., White, N. M., Nandhakumar, Iris S.*.* A novel route to nanostructured bismuth telluride films by electrodeposition. *Electrochem. commun.* **76**, 71–74 (2017).

10. Heo, P., Hagiwara, K., Ichino, R. & Okido, M. Electrodeposition and Thermoelectric Characterization of Bi[sub 2]Te[sub 3]. *J. Electrochem. Soc.* **153**, C213 (2006).

11. Manzano, C. V., Rojas, A. A., Decepida, M., Abad, B., Feliz, Y., Caballero-Calero, O., Borca-Tasciuc, D. A., Martin-Gonzalez, M. Thermoelectric properties of Bi2Te3 films by constant and pulsed electrodeposition. *J. Solid State Electrochem.* **17**, 2071–2078 (2013).

12. Miyazaki, Y. & Kajitani, T. Preparation of Bi2Te3 films by electrodeposition. *J. Cryst. Growth* **229**, 542–546 (2001).

13. Wang, W. L., Wan, C. C. & Wang, Y. Y. Composition-dependent characterization and optimal control of electrodeposited Bi2Te3 films for thermoelectric application. *Electrochim. Acta* **52**, 6502–6508 (2007).

14. Caballero-Calero, O., Díaz-Chao, P., Abad, B., Manzano, C. V., Ynsa, M. D., Romero, Rojo, J. J., Muñoz M., Martín-González, M. S. Improvement of Bismuth Telluride electrodeposited films by the addition of Sodium Lignosulfonate. *Electrochim. Acta* **123**, 117–126 (2014).

15. Yoo, B. Y., Huang, C. K., Lim, J. R., Herman, J., Ryan, M. A., Fleurial, J. P., Myung, N. V. Electrochemically deposited thermoelectric n-type Bi2Te 3 thin films. *Electrochim. Acta* **50**, 4371–4377 (2005).

16. Suresh, A., Chatterjee, K., Sharma, V. K., Ganguly, S., Kargupta, K., Banerjee, D. Effect of pH on structural and electrical properties of electrodeposited Bi 2Te 3 thin films. *J. Electron. Mater.* **38**, 449–452 (2009).

17. Chen, C. L., Chen, Y.Y., Lin, S. J., Ho, J. C., Lee, P. C., Chen, C. D., Harutyunyan, S. R. Fabrication and characterization of electrodeposited bismuth telluride films and nanowires. *J. Phys. Chem. C* **114**, 3385–3389 (2010).

18. Cao, Y., Zeng, Z., Liu, Y., Zhang, X., Shen, C., Wang, X., Gan, Z., Wu, H., Hu, Z. Electrodeposition and Thermoelectric Characterization of (00L)-Oriented Bi2Te3 Thin Films on Silicon with Seed Layer. *J. Electrochem. Soc.* **160**, D565–D569 (2013).

19. Naylor, A. J., Koukharenko, E., Nandhakumar, I. S. & White, N. M. Surfactant-mediated electrodeposition of bismuth telluride films and its effect on microstructural properties. *Langmuir* **28**, 8296–8299 (2012).

20. Li, S., Toprak, M. S., Soliman, H. M. A., Zhou, J., Muhammed, M., Platzek, D., Müller, E. Fabrication of nanostructured thermoelectric bismuth telluride thick films by electrochemical deposition. *Chem. Mater.* **18**, 3627–3633 (2006).

21. Martı́n-González, M. S., Prieto, A. L., Gronsky, R., Sands, T. & Stacy, A. M. Insights into the Electrodeposition of Bi2Te3. *J. Electrochem. Soc.* **149**, C546 (2002).

22. Smith, G. C. *Surface Analysis by Electron Spectroscopy*. (Springer US, 1994). doi:10.1007/978-1-4899-0967-1

23. Anandan, P. *et al.* Tailoring bismuth telluride nanostructures using a scalable sintering process and their thermoelectric properties. 7956–7962 (2014). doi:10.1039/c4ce00837e

24. Bando, H., Koizumi, K., Oikawa, Y., Daikohara, K., Kulbachinskii, V. A., Ozaki, H. The time-dependent process of oxidation of the surface of Bi2Te3 studied by x-ray photoelectron spectroscopy. *J. Phys. Condens. Matter* **12**, 5607–5616 (2000).

25. van der Heide, P. *X-ray Photoelectron Spectroscopy: An introduction to Principles and Practices*. (Wiley, 2012).

26. Noran. Energy-Dispersive X-ray Microanalysis. *Noran Instruments* 68 (1999). doi:10.1007/BF01567579

27. Langford, J. I. & Wilson, A. J. C. Scherrer after sixty years: A survey and some new results in the determination of crystallite size. *J. Appl. Crystallogr.* **11**, 102–113 (1978).

28. Wojdyr, M. Fityk : a general-purpose peak fitting program. *J. Appl. Crystallogr.* **43**, 1126–1128 (2010).

29. Kumar, P., Repaka, D. V. M. & Hippalgaonkar, K. Lithography-free resistance thermometry based technique to accurately measure Seebeck coefficient and electrical conductivity for organic and inorganic thin films. *Rev. Sci. Instrum.* **88**, 125112 (2017).

30. Liu, X. D., Jiang, E. Y. & Zhang, D. X. Electrical transport properties in indium tin oxide films prepared by electron-beam evaporation. *J. Appl. Phys.* **104**, 073711 (2008).

31. Lin, J.-J. & Li, Z.-Q. Electronic conduction properties of indium tin oxide: single-particle and many-body transport. *J. Phys. Condens. Matter* **26**, 343201 (2014).

32. Lin, B. T., Chen, Y. F., Lin, J. J. & Wu, C. Y. Temperature dependence of resistance and thermopower of thin indium tin oxide films. *Thin Solid Films* **518**, 6997–7001 (2010).

33. Li, Z. Q. & Lin, J. J. Electrical resistivities and thermopowers of transparent Sn-doped indium oxide films. *J. Appl. Phys.* **96**, 5918–5920 (2004).

34. Kytin, V. G., Kulbachinskii, V. A., Reukova, O. V., Galperin, Y. M., Johansen, T. H., Diplas, S., Ulyashin, A. G. Conducting properties of In2O3:Sn thin films at low temperatures. *Appl. Phys. A Mater. Sci. Process.* **114**, 957–964 (2014).

35. Guilmeau, E., B́rardan, D., Simon, C., Maignan, A., Raveau, B., Ovono, D., Ovono, D. F. Tuning the transport and thermoelectric properties of In2O3bulk ceramics through doping at In-site. *J. Appl. Phys.* **106**, (2009).

36. Lundstrom, M. Carrier Scattering. in *Fundamentals of Carrier Transport* 271–343 (John Wiley & Sons, Ltd, 2006). doi:10.1002/0470010827.ch9

37. Zalar, S. M. Effect of cumulative annealing on the thermoelectrical parameters of overdoped (Sb, Bi)2 Te3 solid solutions. *Adv. Energy Convers.* **2**, 105–112 (1962).

38. Snyder, G. J. & Toberer, E. S. Complex thermoelectric materials. *Nat. Mater.* **7**, 105–114 (2008).
